# *Arginine Kinase 1* supports energy homeostasis in *Drosophila* flight muscle development

**DOI:** 10.1371/journal.pgen.1012304

**Published:** 2026-09-17

**Authors:** Maria Paula Zappia, Anton Westacott, Hannah Cooke, Rhianna Geary, Libby Travers, Lucia de Castro, Oliver Carty, Maxim V. Frolov

**Affiliations:** Department of Biochemistry and Molecular Genetics, University of Illinois at Chicago, Chicago, Illinois, United States of America; Indian Institute of Science Education and Research Mohali, INDIA

## Abstract

In *Drosophila*, Arginine kinase 1 (Argk1) is involved in maintaining ATP homeostasis during bursts of activity in tissues with high and variable rates of energy turnover such as muscle. However, its role beyond stress conditions is less understood. Argk1 is the sole phosphagen kinase with dynamic expression throughout flight muscle development. Here, we show that at least one of the *Argk1* isoforms localizes to mitochondria in the developing myofibers, and its function is also necessary for proper flight muscle development. Depleting Argk1 specifically in the muscles lead to low ATP level and NAD + /NADH ratio, indicative of defects in energy homeostasis, and results in animal lethality. In the wing disc-associated myoblasts, Argk1 knockdown causes a reduction in cell size without changes in cell cycle progression. Single cell RNA-sequencing (scRNA-seq) revealed that the transcriptomes of undifferentiated and differentiating Argk1-depleted myoblasts are not significantly affected compared to control. Furthermore, based on the marker expression and overall composition of scRNA-seq cell clusters, the early states of myoblasts differentiation are not severely disrupted in Argk1-depleted myoblasts. Nonetheless, Argk1 knockdown severely impacts later stages of muscle development. Remarkably, Argk1-depleted muscles completely lack spontaneous muscle contractions, which are required for proper sarcomere maturation in the formation of the indirect flight muscle. Accordingly, Argk1-depleted muscles showed defects related to sarcomere maturation, and mitochondrial morphogenesis; thus, leading to a severe reduction in muscle growth. Therefore, our data reveal an essential role for Argk1 in flight muscle development, presumably by sustaining local ATP levels to meet the energetic demand to support myofibrillogenesis, muscle growth and proper flight muscle function.

## Author summary

Phosphagen kinases, such as creatine kinases, maintain ATP levels during periods of high energy demand, such as muscle contractions, and thus play a key role in cellular energy homeostasis. In vertebrates, defects caused by the loss of creatine kinase are linked to diseases, including heart failure and muscular dystrophy. In *Drosophila*, the sole gene encoding a phosphagen kinase that is highly expressed in the muscles is Arginine kinase 1 (Argk1). It was previously shown that Argk1 is required to rapidly regenerate ATP to support high metabolic demand during the burst firing in motoneurons. Yet, its function during muscle development remains largely unexplored. Here, we uncover that Argk1 is implicated in the spontaneous contractions of the indirect flight muscles, a process that is associated with proper sarcomere maturation, which is tightly coordinated with mitochondrial morphogenesis. Altogether, our data reveal a role for Argk1 in energy homeostasis at the onset of myofibrillogenesis that is required for proper flight muscle development.

## Introduction

**Fig 1:**
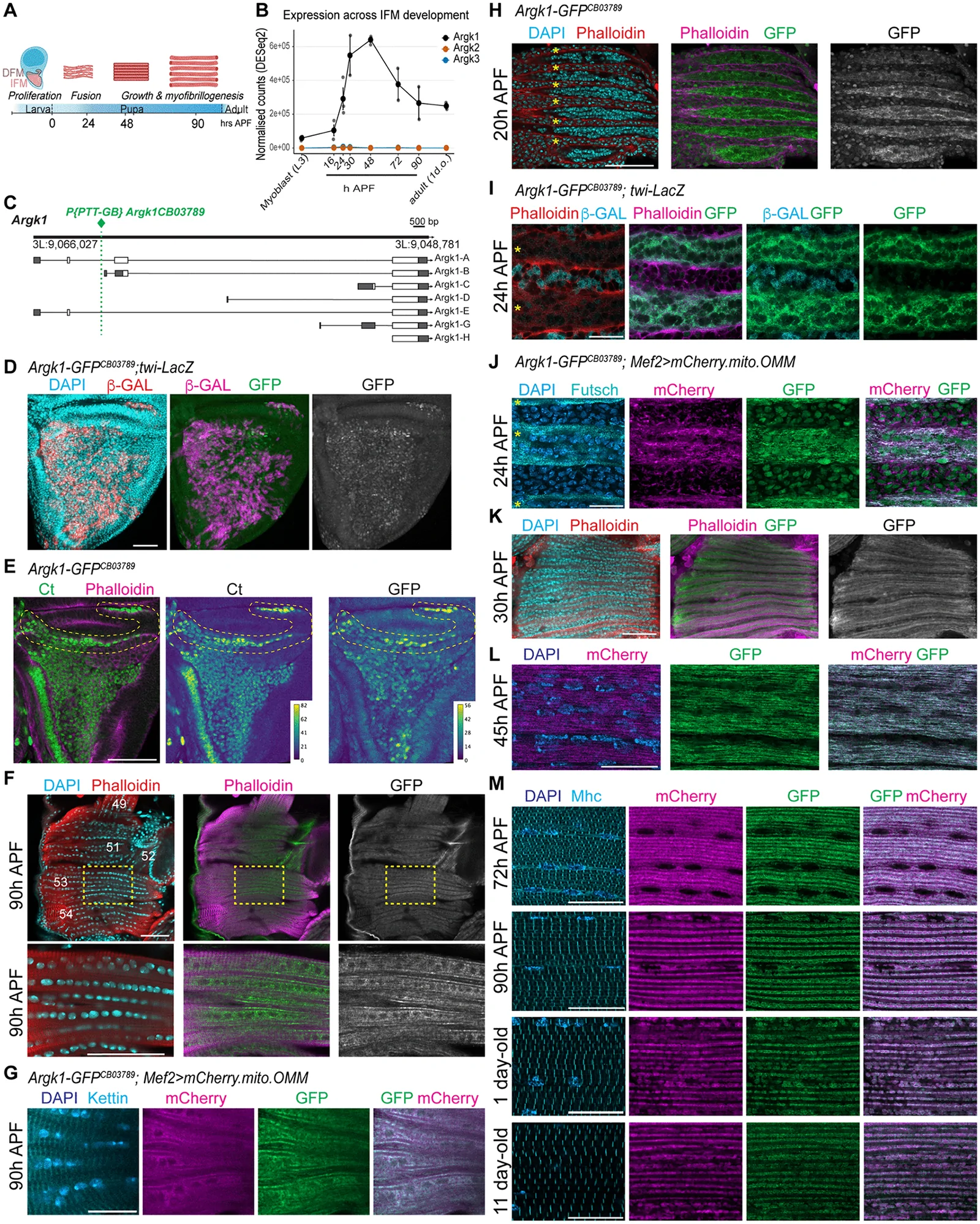
Argk1 is expressed throughout flight muscle development. (**A**) Representation of flight muscle development. Created in BioRender. Zappia, M. P. (2026) https://BioRender.com/xomczyq. (**B**) The mRNA expression of arginine kinase genes, Argk1, Argk2, and Argk3, throughout flight muscle development. Average ± SEM, n = 2–3 replicates (normalized counts measured in [20]). (**C**) Representation of the GFP trap line *Argk1*^CB03789^ inserted in *Argk1* locus (black) showing expected mRNA transcript with coding regions (white) and untranslated regions (grey) and their predicted isoforms [60]. (**D-M**) The GFP trap line *Argk1*^CB03789^ was used to track the expression of Argk1 (Argk1::GFP) in flight muscle development. (**D**) Maximum projection of z-stack from confocal images of third instar larval wing discs labeled with *Twi-LacZ* (β-gal, magenta). (**E**) Confocal single plane image of third instar larval wing discs stained with anti-Ct to label the myoblasts (green). Signal intensity visualized using a Viridis LUT (dark purple to yellow). (**F**) Confocal single plane image of DFM at 90 h APF stained with Phalloidin to mark the myofibers (red and magenta) and with DAPI to label the nuclei (cyan). The DFM muscles are noted, the yellow-dashed box indicates magnified area for the DFM (muscle 53, bottom panel). Anterior to the left. (**G**) Confocal single plane image of DFM at 90 h APF co-stained anti-kettin (cyan) and *mCherry.mito.OMM* (magenta) to label Z-discs and mitochondria, respectively. (**H-L**) Confocal single plane image of forming DLM-IFM at 20 h, 24h, 30 h, and 45h co-stained with Phalloidin (red and magenta), DAPI, *Twi-LacZ* (β-gal, cyan), Futsch (cyan), and *mCherry.mito.OMM* (magenta). Yellow asterisks mark developing myotubes. (**M**) Confocal single plane image of DLM-IFM sagittal sections staged at 72 h and 90 h APF and at 1- and 11-day-old co-stained with Mhc (cyan), DAPI (blue), and *mCherry.mito.OMM* (magenta). Genotypes are: (**D, I**) w; *twi-LacZ*/+; *P{PTT-GB}Argk1*^*CB03789*^/+(**E**-**F**, **H**, **K***) w*; +; *P{PTT-GB}Argk1*^*CB03789*^/+(**G**, **J**, **L**-**M**) *w*; + ; *P{PTT-GB}Argk1*^*CB03789*^/ *Mef2 > mCherry.mito.OMM* Scale (D, F, H, K) 50 µm and (E, G, I, J, L, M) 20 µm.

The *Drosophila* model organism has been extensively used to elucidate the fundamental genetic mechanisms and biological processes underlying muscle development. Somatic fly muscles closely resemble vertebrate skeletal muscles, as both share highly conserved molecular network and the signaling pathways [1,2]. The development of the skeletal muscles follows a precise stepwise process to build fully functional muscles that can support all kinds of locomotion, including flying, walking, jumping and crawling [3]. The *Drosophila* adult skeletal muscles are formed during the second wave of myogenesis later in development [4]. Briefly, the adult muscle precursor (AMP) cells or myoblasts that will give rise to the flight muscles are specified by asymmetric division of the muscle progenitors during embryonic development and remain quiescent until larval stages when they are activated and rapidly proliferate to form a layer of adepithelial cells tightly associated with the wing imaginal discs. Then, the reduction in the expression of the transcription factor Twist and decrease of Notch signaling results in the activation of the myogenic differentiation program [5,6] leading to myoblast fusion, followed by muscle growth, myofibrillogenesis and sarcomere assembly during pupal development (Fig 1A). There are two types of flight muscles, the direct flight muscles (DFM) and the indirect flight muscles (IFM). These two flight muscles show distinct physiology, structure and function [7]. The IFM are fibrillar muscles that power the flight through stretch activated asynchronous contractions allowing very high frequency oscillations, whereas the DFM are tubular muscle fibers with a cross-striated pattern that adjust the angle and rotation of the wings through contraction stimulated by nerve impulse.

Although later stages of muscle formation have been studied in great details, the understanding of early events in myoblast differentiation in the adepithelial layer of the wing disc is limited. One challenge is that the pool of myoblasts is inherently heterogeneous, as cells undergo differentiation and dynamically communicate with the epithelial layer through ligand receptor interactions [8,9]. The advancement of single cell RNA-sequencing helped to begin resolving the heterogeneity of the AMPs [8,10,11]. These cells share some of the features of the vertebrate adult muscle stem cells [12]. The scRNA-seq studies revealed that myoblasts consist of several distinct populations of undifferentiated and differentiating cells and uncovered transcriptional programs underlying diversification of the IFM and DFM at these early stages [10]. Furthermore, single cell experiments have identified genes with unique expression profiles that defined cell clustering, but were not previously associated with muscle development. Whether these genes are merely markers of these cell clusters or whether they may play a role in muscle development remains an open question for most genes. Interestingly, muscle-specific knockdown of one of these genes, *Arginine kinase 1* (*Argk1*), resulted in pupal lethality [10,13], thus suggesting that, at least some of them, may have an important function during muscle development.

*Argk1* belongs to the family of phosphagen kinases and is an analogue of vertebrate creatine kinase. It catalyzes the reversible transfer of the phosphate from adenosine triphosphate (ATP) to Arginine to form phosphoarginine, a high energy molecule that can be used to rapidly generate ATP, which is particularly relevant in tissues with high or fluctuating energy demand, such as muscle, neurons and spermatozoa [14–16]. The primary function of creatine kinase in muscles is to act as a temporal energy buffer and energy transport system. The phosphagen shuttle system connects the sites of ATP productions with the subcellular sites of ATP utilization, thus, offsetting the effects of ATP and ADP diffusion [17]. Indeed, dysfunctional creatine kinase has been associated with muscular dystrophies, inflammatory myopathies and metabolic disorders [18]. Interestingly, each isoform of creatine kinases, which are all encoded by different genes, have tissue-specific expression and defined subcellular localization, cytosol and mitochondrial compartment. In *Drosophila*, the Arginine kinases family consists of four genes, *Argk1*, *Argk2*, *Argk3* and *CG30274* [19], with Argk1 being the sole gene with predominant expression in the muscles [20,21]. The knockdown of Argk1 in motor neurons led to a rapid decline of ATP during axon burst firing that impacts presynaptic energy metabolism [22,23]. Even though extensive research has examined how phosphagen kinases maintain energy homeostasis during different types of stress [24], their functions beyond these conditions are not well understood. Given how little is known about their role during development, we aimed to understand Argk1 function in the developing muscle.

Here, we show that Argk1 is important in *Drosophila* flight muscles development. In proliferating myoblasts, Argk1 is required for cell growth, while at later stages it is needed for proper myofibrillogenesis; along with mitochondrial morphogenesis and muscle growth. Argk1 knockdown results in significant disruption in energy homeostasis, manifested by overall low ATP content and NAD + /NADH ratio. Interestingly, developing Argk1-depleted muscles fail to undergo spontaneous muscle contractions, a process that is associated with building cross-striated muscle fibers in developing abdominal muscles and therefore may explain defects in the maturation of sarcomere and mitochondria. We suggest that when ATP demand exceeds the capacity of glycolysis and oxidative phosphorylation to respond, Argk1 likely sustains local ATP levels. Thus, our work reveals a novel function for Argk1 in supporting energy homeostasis during the spontaneous contractions at the onset of myofibrillogenesis in flight muscle development.

## Results

### Argk1 is expressed throughout the development of both direct and indirect flight muscles

We began by analyzing the expression pattern of Argk1 throughout flight muscle development. First, we used an mRNA-Seq dataset generated at precise time points during flight muscle development [20] and analyzed the expression of all four *Drosophila* genes encoding arginine kinases. Among them, only *Argk1* gene, showed a highly dynamical expression throughout flight muscle development (Fig 1B). Second, we used a GFP trap line *Argk1*^CB03789^ (Argk1::GFP) that was previously used to report the expression of Argk1 *in vivo* [10,22,23]. In this line, the GFP exon is fused in the appropriate reading frame to the endogenous Argk1 protein, thus, likely tagging the two long isoforms Argk1-A and Argk1-E with GFP near the N-terminus (Fig 1C). The wing imaginal discs harboring the myoblasts in the adepithelial layer were dissected from the *Argk*^CB03789^ third instar larva and stained with the myoblasts markers, Cut (Ct) and Twist (Twi). Confocal microscopy reveals strong GFP expression in Twi-positive myoblasts and a much weaker GFP signal in the epithelial cells of the wing disc (Fig 1D). The intensity of Argk1::GFP was much higher in myoblasts giving rise to DFM than IFM as determined by co-staining discs with Ct and plotting the fluorescent intensity profiles for both Ct and Argk1::GFP (Fig 1E, S1 Movie). This is in agreement with published scRNA-seq datasets showing higher expression of Argk1 in myoblasts giving rise to DFM than to IFM [10].

Next, we assessed the expression of Argk1 in mature DFM. The muscles were dissected from pharate-staged Argk1::GFP animals at 90 h APF and the GFP signal was examined. Tissues were stained with Phalloidin and DAPI to visualize developing muscles and nuclei, respectively. Sagittal sections of DFM (muscle 51, 53 and 54) showed strong GFP fluorescence in these muscles while nuclei were largely GFP negative (Fig 1F). Further subcellular localization of Argk1 was examined by co-staining Argk1::GFP with the outer mitochondrial membrane marker, *Mef2 > mCherry.mito.OMM*. We found that Argk1::GFP broadly co-localized with mCherry indicating that Argk1 is likely localized to mitochondria in DFM (Fig 1G).

Since Argk1 is expressed in the myoblasts that give rise to both IFM and DFM (Fig 1D-1E [10]), we also assessed Argk1 expression in both developing and mature IFM. During pupal stages, myoblasts migrate and fuse to form multinucleated myotubes and then undergo myofibrillogenesis and growth (Fig 1A). The dorsal longitudinal muscles (DLM-IFM) were dissected at 20 h, 24 h, 30 h, 45h, 72h and 90 h after pupa formation (APF) representing distinct timepoints in IFM development. Tissues were co-stained with Twi to label myoblasts, Futsch to label myotubes, Mhc to label A-band in the sarcomeric structures, *Mef2 > mCherry.mito.OMM* to label mitochondria, and counterstained with Phalloidin and DAPI [25,26]. We found that Argk1::GFP was strongly expressed in both fusing myoblasts and multinucleated myotubes at 20 h and 24h APF (yellow asterisks), and largely fully localized in mitochondria in the immature myofibers from about 24 h APF onwards throughout IFM development to fully mature muscles (Figs 1H-1M, S1A-S1B). Given that Argk1 was previously found in myofibrils of flight muscles by immunofluorescence [15] we tested the expression pattern of Argk1::GFP in fully formed flight muscles at 1 and 11-days old adults flies. Yet, the GFP signal was mainly detected in mitochondria, likely because the GFP trap line tagged the isoforms Argk1-A and Argk1-E [22,23], and may not report the expression of other Argk isoforms with possible myofibril localization. Thus, our findings support that Argk1 is highly expressed in proliferating myoblasts giving rise to DFM and also show moderate expression in some of the myoblasts giving rise to IFM. Its expression in IFM peaks at mid-pupa development and persists in mitochondria of developing myofibers throughout flight muscle development until adulthood.

### Argk1 is required in adult muscles for animal viability

**Fig 2:**
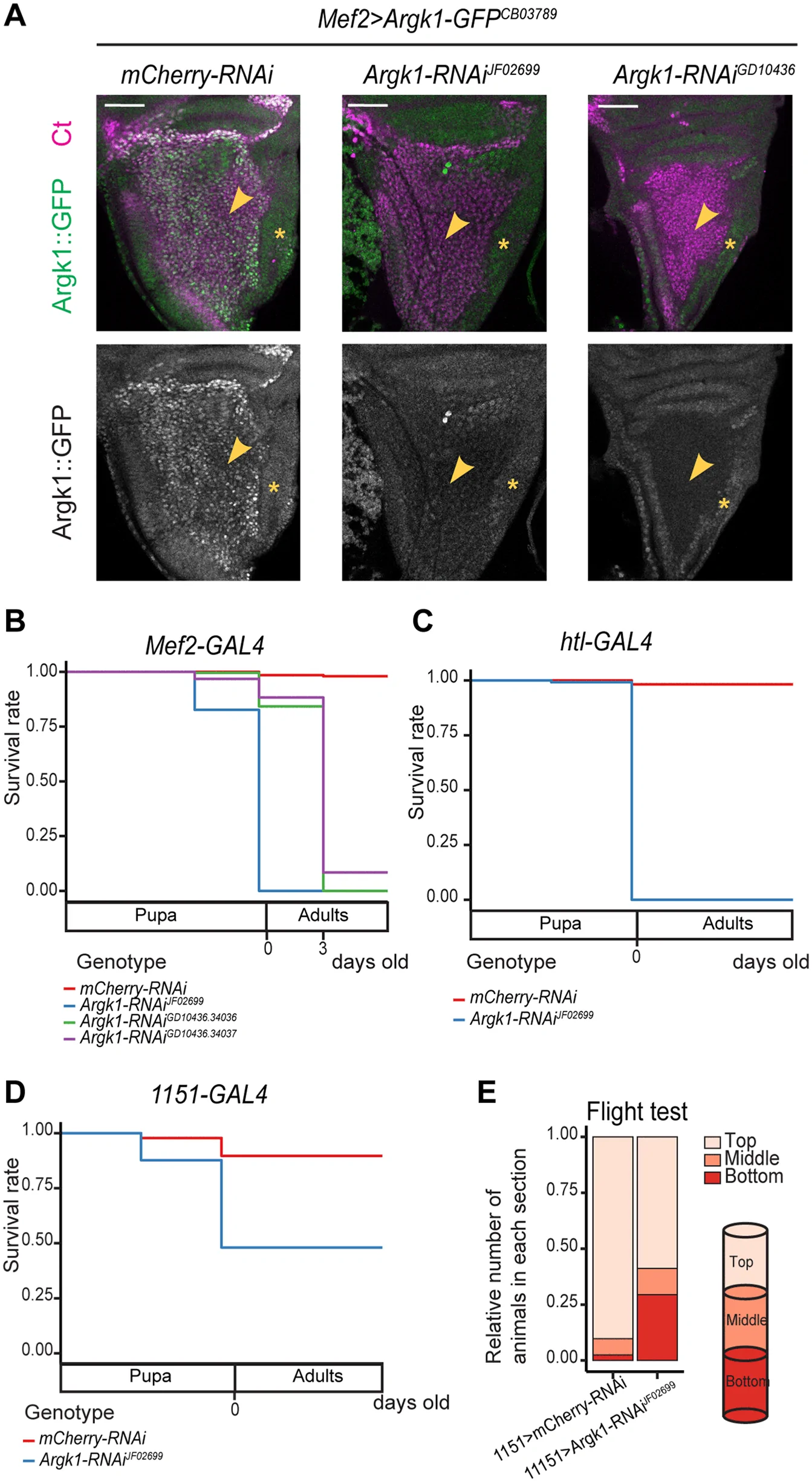
The loss of Argk1 specifically in muscle results in increased lethality during development and flightless animals. Argk1 knockdown using three *UAS-RNAi* lines: transgenes *JF02699* (stock 31959) and *GD10436* with two insertion sites (stocks 34036 and 34037) and compared to control *UAS-mCherry-RNAi*. The drivers used are (**A-B**) *Mef2-GAL4*, (**C**) *htl-GAL4* and (**D-E**) *1151-GAL4*. (**A**) Argk1::GFP expression is lost in the wing discs-associated myoblasts as indicated by the yellow arrowhead in both *Mef2 > Argk1-RNAi* lines, transgene *JF02699* and *GD10436* (stock 34036), while expression persisted in epithelial cells (yellow asterisk). (**B-D**) Kaplan-Meier-like plot to illustrate survival rate of animals through late stages of development, only pupa and adult animals were quantified. Data represent mean of survival rate at each developmental stage scored. (**B**) n = 60 flies per genotype, (**C**) n = 120 flies per genotype, and (**D**) n = 300 flies per genotype. Experiment was repeated N = 2–8 times. (**E**) Flight ability scored by quantifying the percentage of flies landing on section of the cylinder (top, middle and bottom). Stacked bars, n = 17–41 flies /genotype. The ability to flight of the animals that survived in **D** was assessed in **E**. Most males *1151 > Argk1-RNAi*^*JF02699*^ died, and only 17 survived for the flight ability test. Genotypes are: (**A**) w; + ; *Mef2-GAL4*, *P{PTT-GB}Argk1*^*CB03789*^ /*UAS-mCherry-RNAiw; + ; Mef2-GAL4*, *P{PTT-GB}Argk1*^*CB03789*^/*UAS-Argk1-RNAi*^*JF02699*^ *w; + ; Mef2-GAL4*, *P{PTT-GB}Argk1*^*CB03789*^/*UAS-Argk1-RNAi* ^*GD10436. 34036*^ (**B**) w; + ; *Mef2-GAL4*/*UAS-mCherry-RNAi* w; + ; *Mef2-GAL4*/*UAS-Argk1-RNAi*^*JF02699*^ w; + ; *Mef2-GAL4*/*UAS-Argk1-RNAi* ^*GD10436.34036*^ w; + ; *Mef2-GAL4*/*UAS-Argk1-RNAi* ^*GD10436.34037*^ (**C**) w; + ; *htl-GAL4*/*UAS-mCherry-RNAi* w; + ; *htl-GAL4*/*UAS-Argk1-RNAi*^*JF02699*^ (**D-E**) *1151-GAL4*; +; *UAS-mCherry-RNAi/+1151-GAL4*; + ; *UAS-Argk1-RNAi*^*JF02699*^*/+*.

To confirm that GFP accurately reflects the endogenous expression of Argk1, we performed RNAi experiments to knockdown Argk1 in myoblasts using the *Mef2-GAL4* driver and two independent *UAS-Argk1-RNAi* transgenes: *JF02699* and *GD10436*, the latter was shown to efficiently deplete Argk1 in the adult brain [27]. In both cases, the GFP signal was lost specifically in myoblasts labeled with anti-Ct antibody (yellow arrowhead), while the GFP expression was unaffected in the epithelial cells of the wing disc (Ct-negative cells, yellow asterisk, Fig 2A). Thus, we concluded that Argk1::GFP accurately reflects the endogenous expression of, at least, two isoforms of Argk1 and the *UAS-Argk1-RNAi* transgenes efficiently deplete Argk1 in the wing discs-associated myoblasts at third instar larva.

To explore the requirement for Argk1 in muscle development we used different muscle-specific GAL4 drivers that are expressed at different time points and in different muscles. The depletion of Argk1 with the pan-muscle *Mef2-GAL4* driver, which is expressed in all muscles throughout development, using the *UAS-Argk1-RNAi* line *JF02699* led to lethality at pharate stage (Fig 2B). The knockdown of Argk1 using the second *UAS-Argk1-RNAi* transgene *GD10436* also resulted in lethality, albeit at a later time point, as most animals died within 3 days post-eclosion (Fig 2B), which is in agreement with previous reports [13].

The *UAS-Argk1-RNAi* transgene *JF02699* that gave stronger phenotype than *GD10436* was further tested with two additional GAL4 drivers, *htl-GAL4* and *1151-GAL4*. The *htl-GAL4* is expressed in the mesoderm and muscle lineages, including the adult muscle precursors [8,28]. Depletion of Argk1 with *htl-GAL4* resulted in lethality at the pharate stage (Fig 2C), similar to what we found with *Mef2-GAL4*. The other GAL4 driver, *1151-GAL4*, is expressed in myoblasts giving rise to adult skeletal muscles, but it is not expressed in larval muscle lineage [5]. The knockdown of Argk1 with *1151-GAL4* resulted in partial lethality as half of the animals died at pharate stage (Fig 2D). Notably, the surviving animals showed defective muscle function, since more than the third of flies failed to reach the top of the cylinder in the flight test (Fig 2E).

We concluded that Argk1 is essential in muscle development, as its knockdown using a pan-muscular as well as an adult-muscle specific GAL4 drivers resulted in lethality and decreased muscle function, respectively.

### Argk is required for the development of both direct and indirect flight muscles

**Fig 3:**
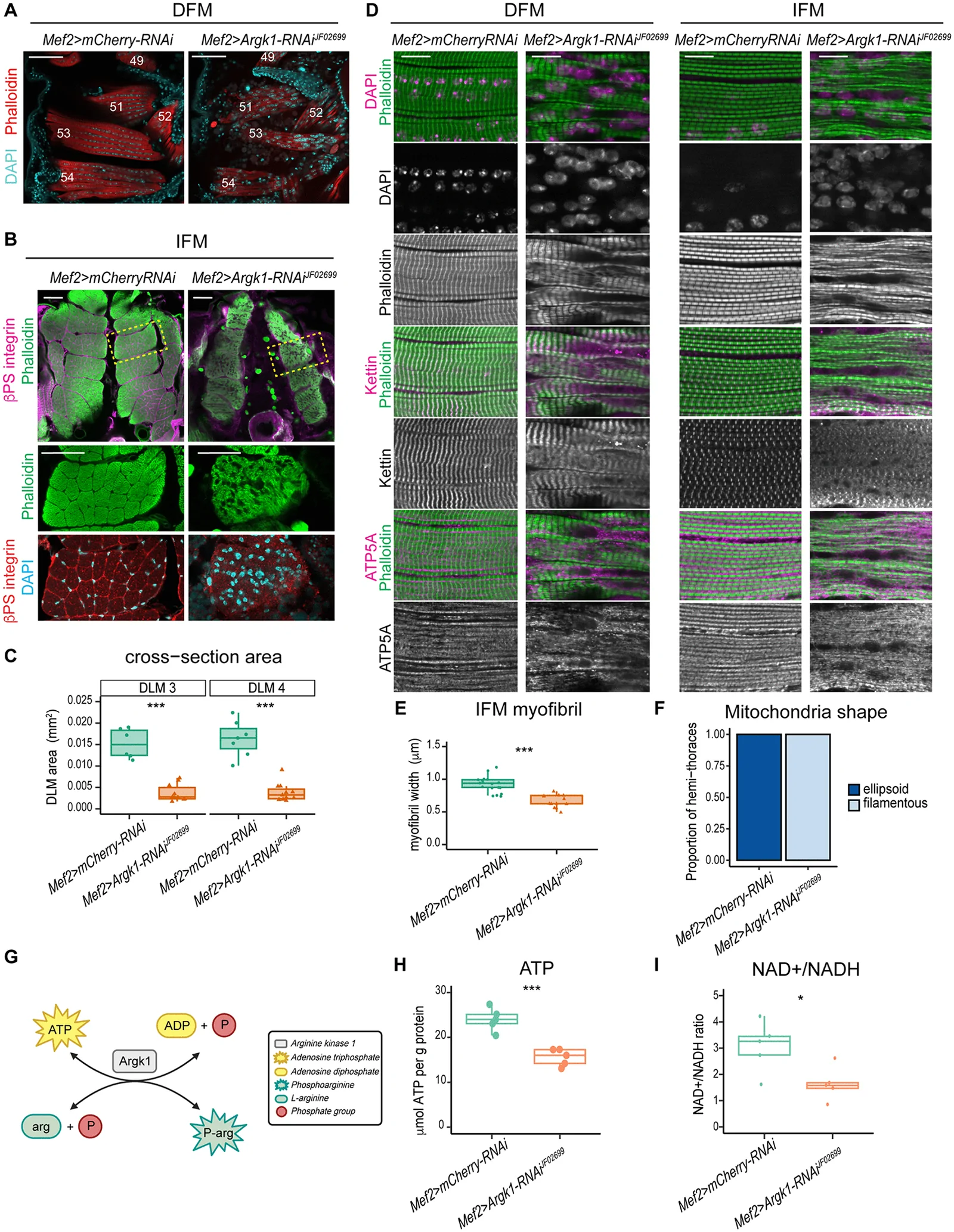
Argk1 knockdown with *Mef2-GAL4* results in severe defects in both IFM and DFM. (**A-B, D**) The structure of the DFM and IFM examined in *Mef2 > Argk1-RNAi*^*JF02699*^ and control *Mef2 > mCherry-RNAi* animals staged at 90h APF. (A) Confocal single plane images of DFM in a sagittal view. Hemithorax sections stained with Phalloidin (red) and DAPI. DFM are numbered in white as in [61]. Anterior is to the left. (B) Confocal single plane images of IFM in a transverse view of the DLM-IFM. Sections stained with Phalloidin (green), DAPI (cyan) and βPS-integrin (magenta and red). Yellow-dashed box indicates magnified area for muscle 3 from DLM-IFM (bottom panel). Dorsal is up. (C) Quantification of the cross-section area for muscle 3 and 4 from DLM-IFM per animal. N = 6–11 thoraces/genotype, Wilcoxon rank-sum test, Bonferroni-corrected for multiple comparison, *** p < 0.001. (D) Confocal single plane images of DFM (muscle 53, left panel) and DLM-IFM (right panel) in a sagittal view. Hemithorax sections stained with Phalloidin (green), DAPI (magenta), anti-Kettin (magenta) and anti-ATP5A (magenta). (E) Quantification of myofibril width for the DLM-IFM per animal. N = 13–18 thoraces/genotype, Welch Two Sample t-test, *** p < 0.001. (F) Blind quantification of the number of hemi-thoraces displaying either proper morphology of mitochondria (ellipsoid) or altered mitochondrial shape (filamentous) from DLM-IFM. Stacked bars. N = 18–20 hemithoraces/genotype. N = 2 independent experiments. (G) Simplified representation of the phosphagen system, in which Argk1 transfer a phosphate from ATP to arginine and make ADP and phosphoarginine in a reversible manner. Created in BioRender. Zappia, M. P. (2026) https://BioRender.com/6zyn1a4. (H) Luminescence-based ATP measured on extracts harvested from *Mef2 > mCherry-RNAi* and *Mef2-Argk1-RNAi*^*JF02699*^ pharate animals staged at 90h APF. Values were normalized to total protein content. n = 5 samples/genotype, Welch Two Sample t-test, *** p < 0.001. N = 2 independent experiments. One representative experiment is shown. (I) Fluoremetric NAD + /NADH ratio measured on extracts harvested from *Mef2 > mCherry-RNAi* and *Mef2-Argk1-RNAi*^*JF02699*^ pharate animals staged at 90h APF. n = 5 samples/genotype, Welch Two Sample t-test, * p < 0.05. N = 2 independent experiments. One representative experiment is shown. All box plot shows median (middle), interquartile range (box), 1.5x the interquartile range (whiskers), and individual data (points). Scale (**A-B**) 50 µm, and (**D**) 10 µm. Genotypes are: w; + ; *Mef2-GAL4*/*UAS-mCherry-RNAi* w; + ; *Mef2-GAL4*/*UAS-Argk1-RNAi*^*JF02699*^.

The dynamic expression of Argk1 in flight muscles (Fig 1B) and the results of knocking down its expression with an adult muscle specific-GAL4 driver (Fig 2D-2E) suggest that Argk1 plays an important role in flight muscle formation. To determine how the loss of Argk1 function affects flight muscle development, we examined the structure of DFM and IFM in animals expressing the *UAS-Argk1-RNAi* ^*JF02699*^ transgene under the control of *Mef2-GAL4* (*Mef2 > Argk1-RNAi*). Transverse and sagittal sections of *Mef2 > Argk1-RNAi* and control pharate adults were stained with Phalloidin and anti β-PS-integrin antibody to visualize muscle structure (Fig 3A-3B). Notably, the *Argk1*-depleted DFM and IFM were reduced in size. Quantifications confirmed that the cross-sectional area of muscle 3 and 4 from DLM-IFM were statistically significantly smaller than in wild type control (Fig 3C). These results were validated with another *UAS-Argk1-RNAi* line *GD10436* that produced similar, but milder phenotype (S2A-S2B Fig). The milder phenotype induced by *GD10436* was consistent with the results shown above (Fig 2B).

To visualize the overall sarcomere organization and mitochondrial morphology of Argk1-depleted muscles, sagittal sections of DFM and IFM were stained with Phalloidin to label the myofibrils, anti-Kettin antibody to label the Z-discs and anti-ATP5A antibody to visualize mitochondria. In the IFMs of control pharates, mitochondria are tightly packed between the myofibrils and form a tubular and elongated mitochondrial network, while the Z-discs in the sarcomere are visualized as regular, evenly-spaced stripes along the myofibrils (Fig 3D). Severe defects in myofibril assembly and a significant reduction in myofibril width were found in IFM of *Mef2 > Argk1-RNAi* pharate adults (Fig 3D, right panel, Fig 3E), thus suggesting a defect in the process of myofibrillogenesis [20]. Strikingly, the mitochondrial network was highly abnormal in Argk1-depleted muscles as it no longer exhibited the ellipsoid shape found in the control (Fig 3D). A blind quantification of mitochondrial network shape, categorized as either ellipsoid or filamentous, confirmed that the depletion of Argk1 resulted in significant alterations in mitochondrial morphology (Fig 3F). Similarly, alterations in the morphology of both mitochondria and myofibril were also found in the DFMs of the Argk1-depleted muscles compared to control (Fig 3D, left panel). The phenotypes were confirmed with the other *UAS-Argk1-RNAi* line *GD10436* (S2C Fig) by quantifying defects in both sarcomere length and myofibril width (S2D-S2E Fig). The defects in mitochondria structure were less severe (S2C, S2F-S2G Fig), which is consistent with the weaker phenotypes caused by *GD10436* transgene in other tests (Figs 2, S2). Overall, our findings further support the requirement of Argk1 during the development of both flight muscles, DFM and IFM, as muscle growth, mitochondria expansion and myofibrillogenesis were impaired. Given the interplay and coordination between myofibril morphogenesis and mitochondrial dynamics [29], the abnormal shape of the mitochondrial network could be due to defects in the properly forming myofibrils in the Argk1-depleted muscle.

### Argk1 knockdown in the muscle affects energy homeostasis

Given that Argk1 is involved in buffering ATP content (Fig 3G), we explored the impact of Argk1 knockdown on energy homeostasis by examining the ATP level in Argk1-depleted muscles. Extracts from animals staged at pharate stage were prepared to measure the total ATP content by a luminescence-based ATP assay, which was then normalized to the total protein content to account for the differences in muscle mass. Notably, the level of ATP was significantly reduced in *Mef2 > Argk1-RNAi* animals relative to control (Fig 3H). Additionally, we measured NAD + /NADH ratio in *Mef2 > Argk1-RNAi* and control animals staged at pharate stage using a sensitive fluorescence-based quantification assay. Consistently, the NAD + /NADH ratio was significantly lower in the Argk1-depleted muscles compared to the control (Fig 3I), thus indicating an increase in the reductive state.

Overall, these results demonstrate that Argk1 depletion results in severe metabolic abnormalities including low ATP level and altered NAD + /NADH ratio.

### Argk1 impacts cell size of wing discs-associated myoblasts

**Fig 4:**
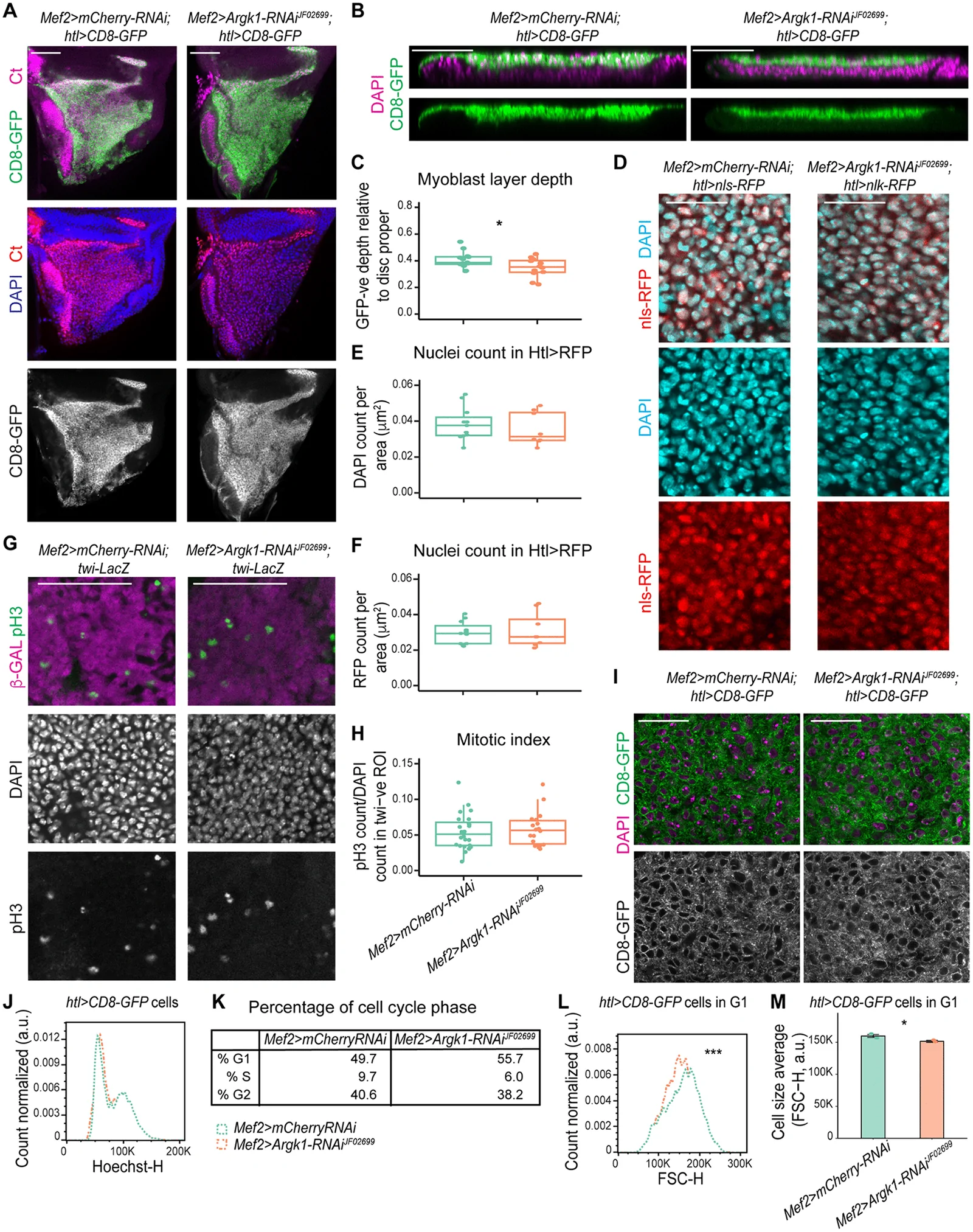
Knockdown of Argk1 reduces cell size but does not affect cell cycle progression. The wing discs-associated myoblasts of the third instar larval wing discs *Mef2 > Argk1-RNAi*^*JF02699*^ compared to control *Mef2 > mCherry-RNAi*. (A) Confocal single plane images of wing discs stained with anti-Ct (red and magenta), *htl-LexA*; *lexO-CD8-GFP* (green) to label the myoblasts and DAPI (blue). (B) XZ orthogonal view of wing discs centered in the notum stained with *htl-LexA*; *lexO-CD8-GFP* (green) and DAPI (magenta). (C) Quantification of the depth of the myoblast layer from XZ orthogonal view in B. Ratio between depth of the GFP-positive cells relative to whole disc per disc. n = 12–13 discs per genotype, two-tailed Student’s t- test, p < 0.05. N = 2 independent experiments. (D) Confocal single plane images of wing discs labeled with *htl-LexA*; *LexO-nls-RFP* (red) to mark the myoblasts and DAPI (blue). (E-F) Quantification of the number of myoblasts per area using (E) DAPI and (F) *htl > nls-RFP*. n = 8–11 discs/genotype, Wilcoxon rank-sum test, p < 0.05. N = 3 independent experiments. (G) Confocal single plane images of wing discs stained with anti-pH3 (green) to label mitotic cells, *twi-LacZ* (magenta) to mark the myoblasts and DAPI. (H) Quantification of the number of pH3-positive cells relative to the number of nuclei (DAPI count) within the Twist positive area. n = 18–24 discs/genotype, Wilcoxon rank-sum test, p < 0.05. N = 3 independent experiments. (I) High magnification, confocal single plane images of wing discs labeled with *htl-LexA*; *lexO-CD8-GFP* (green) and DAPI (magenta). (J) Histogram of DNA content vs myoblast count. Cells sorted by flow cytometry using *htl > CD8-GFP* and normalized to unit area, n = 7168 and 7894 cells/genotype. N = 2 independent experiments. (K) Percent of cells in G1, S and G2 from (J) as determined by Dean-Jett-Fox model. N = 2 independent experiments. (L) Histogram of relative cell size as measured by forward scatter vs myoblast counts. Cells selected in G1 from (J) normalized to unit area, n = 3403 and 4282 cells per genotype. Welch Two Sample t-test, *** p < 0.001. (M) Bar plot showing average cell size normalized to unit area per experiment measured by forward scatter for myoblast cells in G1 from (J). N = 2 independent experiments. One-side paired t-test, * p < 0.05. All box plot shows median (middle), interquartile range (box), 1.5x the interquartile range (whiskers), and individual data (points). Scale (A, B, G) 50 µm, and (D, I) 20 µm. Genotypes are: (A-C, I-M) w; *lexO-CD8-GFP/ +* ; *htl-LexA, Mef2-GAL4*/*UAS-mCherry-RNAi* w; *lexO-CD8-GFP/ +* ; *htl-LexA, Mef2-GAL4*/*UAS-Argk1-RNAi*^*JF02699*^ (D-F) w; *lexO-nls-RFP/ +* ; *htl-LexA, Mef2-GAL4*/*UAS-mCherry-RNAi* w; *lexO-nls-RFP/ +* ; *htl-LexA*, *Mef2-GAL4*/*UAS-Argk1-RNAi*^*JF02699*^ (G-H) w; *twi-LacZ/ +* ; *Mef2-GAL4*/*UAS-mCherry-RNAi*w; *twi-LacZ/ +* ; *Mef2-GAL4*/*UAS-Argk1-RNAi*^*JF02699*^.

Next, we investigated whether Argk1 has a role at the early stages of flight muscle development, specifically during proliferation of wing disc-associated myoblasts. Examining overall distribution of myoblasts in the *Mef2 > Argk1-RNAi* wing discs by staining with both anti-Cut antibodies and *htl-lexA > lexO-CD8-GFP* reporter [30] did not reveal severe changes compared to the control (Fig 4A). However, we noticed a small reduction in the depth of the layer of Argk1-depleted myoblasts normalized to the depth of the disc proper compared to control (Fig 4B-4C). The reduced depth of the myoblast layer could be due to a reduction in either the number of cells and/or in the cell size. To examine the former possibility, we measured the number of myoblasts per area. The myoblasts nuclei were labeled with *htl > nls-RFP*, and both the number of DAPI and RFP positive nuclei per image were counted in an automated manner using the ImageJ software (Fig 4D). No significant differences were observed between *Mef2 > Argk1-RNAi* and the control (Fig 4E-4F). In addition, we assessed cell proliferation by staining wing imaginal discs with a mitotic marker, anti-phospho-histone 3 (pH3) antibody. To distinguish the pH3 positive myoblasts from pH3 positive epithelial cells, *twi-LacZ* was used to label the myoblasts (Fig 4G). The number of pH3-positive myoblasts relative to the number of cells per image did not show significant change between Argk1-depleted and control myoblasts (Fig 4H).

So far, our data disfavor a defect in cell cycle proliferation or myoblast number in the Argk1-depleted myoblasts, thus, raising the possibility of a defect in cell size. However, the high variation in cell size [31] precluded us from directly addressing this question by fluorescent microscopy (Fig 4I). That is the reason why we pivoted to flow cytometry to accurately determine the cell size of Argk1-depleted myoblasts. To quantify cell cycle phases along with cell size, the wing discs from *Mef2 > mCherry-RNAi;htl > CD8-GFP* and *Mef2 > Argk1-RNAi;htl > CD8-GFP* animals were dissociated into single cell suspension, stained with Hoechst and sorted by Fluorescence-Activated Cell Sorting (FACS) to determine DNA content and forward light scatter (FSC), a measurement of cell size. The myoblasts were distinguished from epithelial cells of the wing disc by the presence of GFP while their cell cycle profile was determined based on the intensity of Hoechst fluorescence that reflects the DNA content (Fig 4J). The Dean-Jett-Fox model was fitted to estimate the percentage of G1 (or 2C), S and G2 (4C) cells for the myoblasts. The percentage of G1, S and G2 cells were comparable between control and Argk1-depleted myoblasts (Fig 4K), which is consistent with normal mitotic index in *Mef2 > Argk1-RNAi,* as determined by immunofluorescence (Fig 4G-4H). To account for cell size variation due to cell cycle phase only cells undergoing G1 were further analyzed. Remarkably, the cell size of *Mef2 > Argk1-RNAi* myoblasts was significantly smaller than that of control cells as revealed by the measurement of forward scatter (FSC) of the G1 cells (Fig 4L), across independent experiments (Fig 4M). These results suggest that although Argk1-depleted myoblasts proliferate normally, their size is slightly smaller, which may explain the reduction in the depth of the myoblasts layer in the *Mef2 > Argk1-RNAi* wing imaginal discs (Fig 4B-4C). These findings support the idea that Argk1 is important for cell growth during myoblast proliferation at the onset of flight muscle development. However, it is not required for cell cycle progression.

### The Argk1 depletion does not alter the identity of the wing discs-associated myoblasts

**Fig 5:**
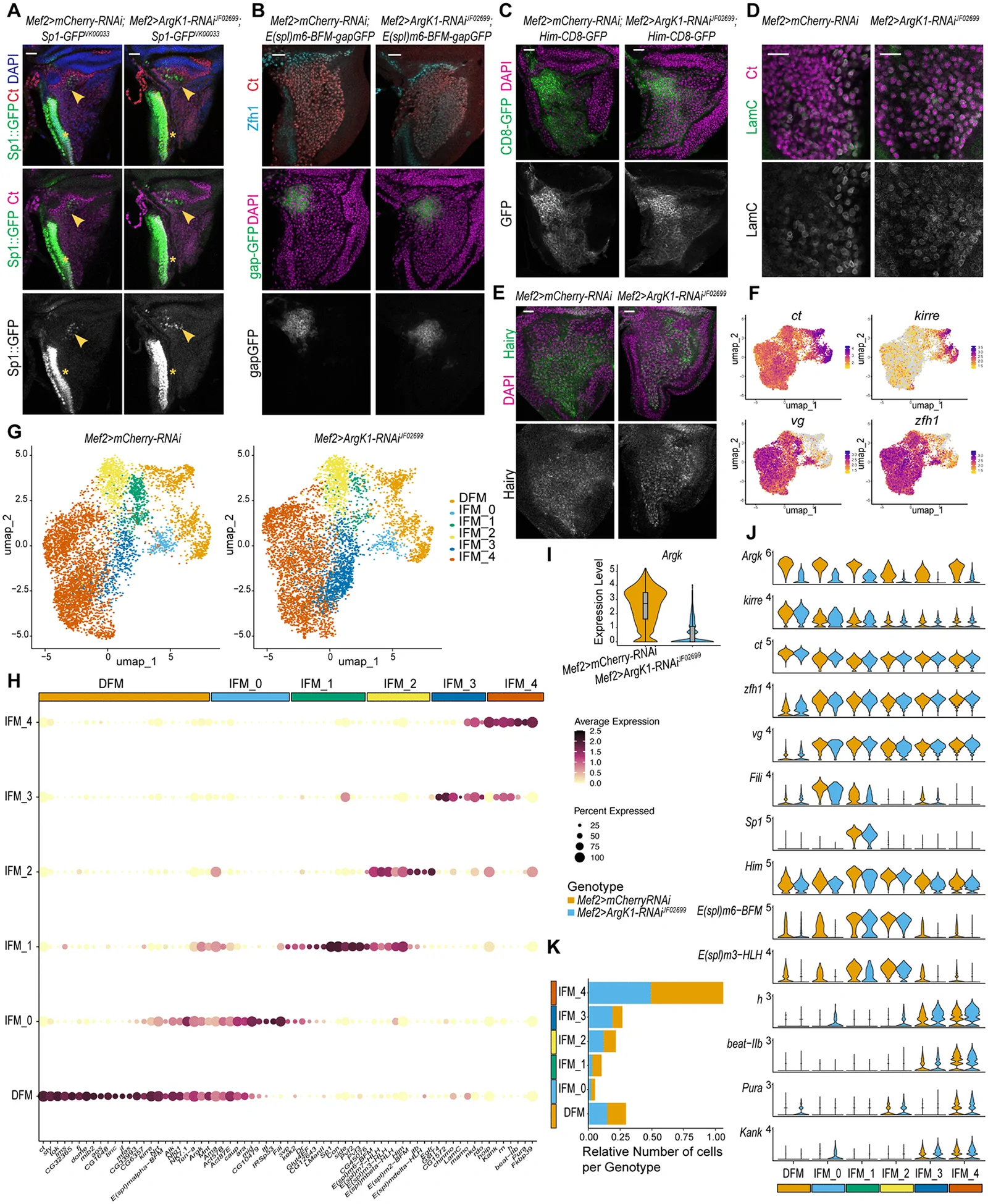
Knockdown of Argk1 does not alter the overall identity of the undifferentiated and differentiating myoblasts. (**A-E**) Confocal single plane images of *Mef2 > mCherry-RNAi* and *Mef2 > Argk-RNAi*^*JF02699*^ third instar larval wing discs labeled with (**A**) *Sp1-GFP*^*VK00033*^ (green), (**B**) *E(spl)m6-BFM-gapGFP* (green), (**C**) *Him-CD8-GFP* (green), and stained with (**D**) anti-LamC (green), (**E**) anti-Hairy (green), and co-stained with (**A-B,D**) anti-Ct (magenta), (**B**) anti-Zfh1 (cyan), and (**A,C,E**) DAPI (blue and magenta). (F) Average expression level of the genes used to assign DFM and IFM clusters. Cells colored by the expression of *ct*, *kirre*, *vg*, and *zfh1*. (G) Two-dimensional UMAP representation of both the combined *Mef2 > Argk1-RNAi*^*JF02699*^ (5,681 myoblasts) and *Mef2 > mCherry-RNAi* (5,808 myoblasts) from third instar larval wing discs datasets, colored by cell cluster and split by genotype. (H) Dot plots showing the expression levels of the top known markers for each cluster and selected genes from the top markers list identified in this dataset. Color intensity shows the average expression level normalized and scaled. The size of the dot represents the fraction of cells within a cluster expressing each gene. (I) Violin plot showing the expression of *Argk1* for the combined datasets *Mef2 > mCherry-RNAi* (yellow) and *Mef2 > Argk1-RNAi*^*JF02699*^ (blue). (J) Stacked violin plots representing the distribution of expression of myoblast markers across clusters of the combined datasets. Genes include *Argk1*, *kin of irre* (*kirre*), *cut* (*ct*), *Zn finger homeodomain 1* (*zfh1*), *vestigial* (*vg*), *Fili* (*Fish-lips*), *Sp1*, *Holes in muscle* (*Him*), *Enhancer of split m6* (*E(spl)m6-BFM*), *Enchancer of split m3* (*E(spl)m3-HLH*), *hairy* (*h*), *beaten path IIb* (*beat-IIb*), *Pura* (*Puratrophin-1-like*), *Kank*. Data were split by genotype, *Mef2 > mCherry-RNAi* (yellow) a*nd Mef2 > Argk-RNAi*^*JF02699*^ (blue). (K) Total number of cells per cluster per genotype relative to the total number of myoblasts per genotype of the combined datasets *Mef2 > mCherry-RNAi* (yellow) and *Mef2 > Argk1-RNAi*^*JF02699*^ (blue). Scale 20 µm. Genotypes are: (**A**) w; +; *Sp1-GFP*^*VK00033*^/+, *Mef2-GAL4*/*UAS-mCherry-RNAi*w; +; *Sp1-GFP*^*VK00033*^/+, *Mef2-GAL4/UAS-Argk1-RNAi*^*JF02699*^ (**B**) w; *E(spl)m6-BFM-gapGFP*/ + ; *Mef2-GAL4*/*UAS-mCherry-RNAi* w; *E(spl)m6-BFM-gapGFP*/ + ; *Mef2-GAL4/UAS-Argk1-RNAi*^*JF02699*^ (**C**) w; *Him-CD8-GFP*/ + ; *Mef2-GAL4*/*UAS-mCherry-RNAi* w; *Him-CD8-GFP*/ + ; *Mef2-GAL4/UAS-Argk1-RNAi*^*JF02699*^ (**D-K**) w; + ; *Mef2-GAL4*/*UAS-mCherry-RNAi* w; + ; *Mef2-GAL4/UAS-Argk1-RNAi*^*JF02699*^.

The single-cell transcriptomics atlas of the wing disc-associated myoblasts identified several transcriptionally distinct cell clusters reflecting the transition from undifferentiated to differentiating cell states [8,10]. To explore whether *Argk1* knockdown alters the progression of myoblasts through these differentiation states, we examined the expression of cell cluster-specific markers in Argk1-depleted myoblasts. According to the reference atlas [10], *Sp1, Him* and *E(spl)m6-BFM* are highly expressed in the undifferentiated clusters IFM_1 and IFM_2, while Lamin C and Hairy are markers for the differentiating clusters IFM_3 and IFM_4. The expression of *Sp1::GFP*, *Him-CD8-GFP* and *E(spl)m6-BFM-gapGFP* reporters in *Mef2 > Argk1-RNAi* and *Mef2 > mCherry-RNAi* wing discs was examined by immunofluorescence (Fig 5A-5C). Anti-Ct and anti-zfh1 antibodies were used to label DFM and IFM myoblasts. No apparent changes in the expression of these markers were found. Similarly, the staining with antibodies against Lamin-C (anti-LamC) and Hairy (anti-H) revealed no significant defects in their pattern of expression (Fig 5D-5E).

Transcriptional changes orchestrate and drive the morphological transitions that occur during muscle development [20]. To further explore the progression of differentiation in Argk1-depleted myoblasts and complement the results of marker analysis described above, we performed scRNA-seq. The wing discs of *Mef2 > Argk1-RNAi* and *Mef2 > mCherry-RNAi* third instar larva were dissociated into single cell suspensions, and the transcriptomes of individual cells were determined using the 3’ Gene Expression assay (10X Genomics). The data were processed using Seurat v5 package [32] to cluster cells based on gene expression similarity. After filtering poor quality cells and applying Principal Component Analysis, the non-linear dimensionality reduction algorithm, UMAP, was used for visualization of cell clusters. A total of 11,489 myoblasts across two genotypes and two replicates each were selected for downstream analysis (S1 Data). Based on the differential expression of the canonical DFM and IFM marker genes, *ct*, *vg, zfh1 and kirre*, we identified clusters giving rise to DFM and IFM (Fig 5F).

Before comparing the transcriptomes of wild type and Argk1-depleted myoblasts, we first annotated the cell clusters using a panel of myoblast markers derived from previous scRNA-seq studies [8,10] (Fig 5G-5H, S2 Data). Notably, we identified IFM_1 based on the high expression of the biomarkers *Sp1*, *Con* and *side*, IFM_2 based on *E(spl)m6-BFM*, *E(spl)m3-HLH*, *E(spl)m2-BFM*, *E(spl)m7-HLH*, and *Him*, IFM_3 based on *chinmo*, *LamC* and *mamo*, and IFM_4 based on *h* and *Fkbp39*. Additionally, we identified a new cluster, IFM_0, showing high expression of the markers *Itl*, *Fili*, *svp*, *IRSp53*, and *mid* (Fig 5G-5H). Interestingly, IFM_0 share many features with DFM and IFM_1. Furthermore, we identified novel markers for each cluster with a selected gene panel visualized by a dot plot (Fig 5H, S2 Data). Finally, we extended the list to the top 40 cluster specific marker genes and visualized them by a hierarchical clustered heatmap (S3A Fig, S3, S4 Datas).

To determine whether the depletion of Argk1 affects the myoblasts across undifferentiated and differentiating clusters, we compared the single cell transcriptomes between *Mef2 > Argk1-RNAi* (5,681 myoblasts) and the control *Mef2 > mCherry-RNAi* (5,808 myoblasts). In agreement with the *in vivo* results (Fig 2A), the expression of Argk1 was significantly reduced in the Argk1-depleted myoblasts (Fig 5I-5J, S5 Data). In agreement with the earlier work [10], and the *Argk1:GFP* reporter expression (Figs 1D-1E, 2A), *Argk1* is highly expressed in DFM, IFM_0 and IFM_1 clusters. The distribution of myoblasts for each genotype showed no severe difference in the contribution of Argk1-depleted myoblasts across cell clusters (Fig 5K). This was consistent across the two replicate samples (S3B-S3C Fig, S1 Table). Next, we compared the expression of a panel of known marker genes between Argk1-depleted and control myoblasts across all IFM clusters and the DFM cluster (Fig 5J, S5 Data). Overall, no significant changes were found in the expression of DFM markers (*kirre* and *ct*) and IFM markers (*zfh1* and *vg*), as well as markers for undifferentiated IFM clusters, (Fili, *Sp1*, *Him*, and for differentiating IFM clusters (*h*, *beat-IIb*, *Pura*, and *Kank*); albeit small changes were found for *E(spl)m6-BFM*, *E(spl)m3-HLH* in IFM_0. In fact, pseudobulk analysis using DE-seq2 showed that the expression of very few genes was significantly different between *Mef2 > Argk1-RNAi* and *Mef2 > mCherry-RNAi* either across individual myoblast clusters or when grouping them together (S5, S6 Datas, S4A-S4B Fig).

Thus, the depletion of Argk1 does not affect the identity of myoblasts across undifferentiated and differentiating states nor the expression or localization of the well-known myoblast markers. This suggests that the early stages of differentiation are not severely disrupted in Argk1-depleted myoblasts.

### Argk1-depleted muscles fail to undergo spontaneous contractions during pupal development

**Fig 6:**
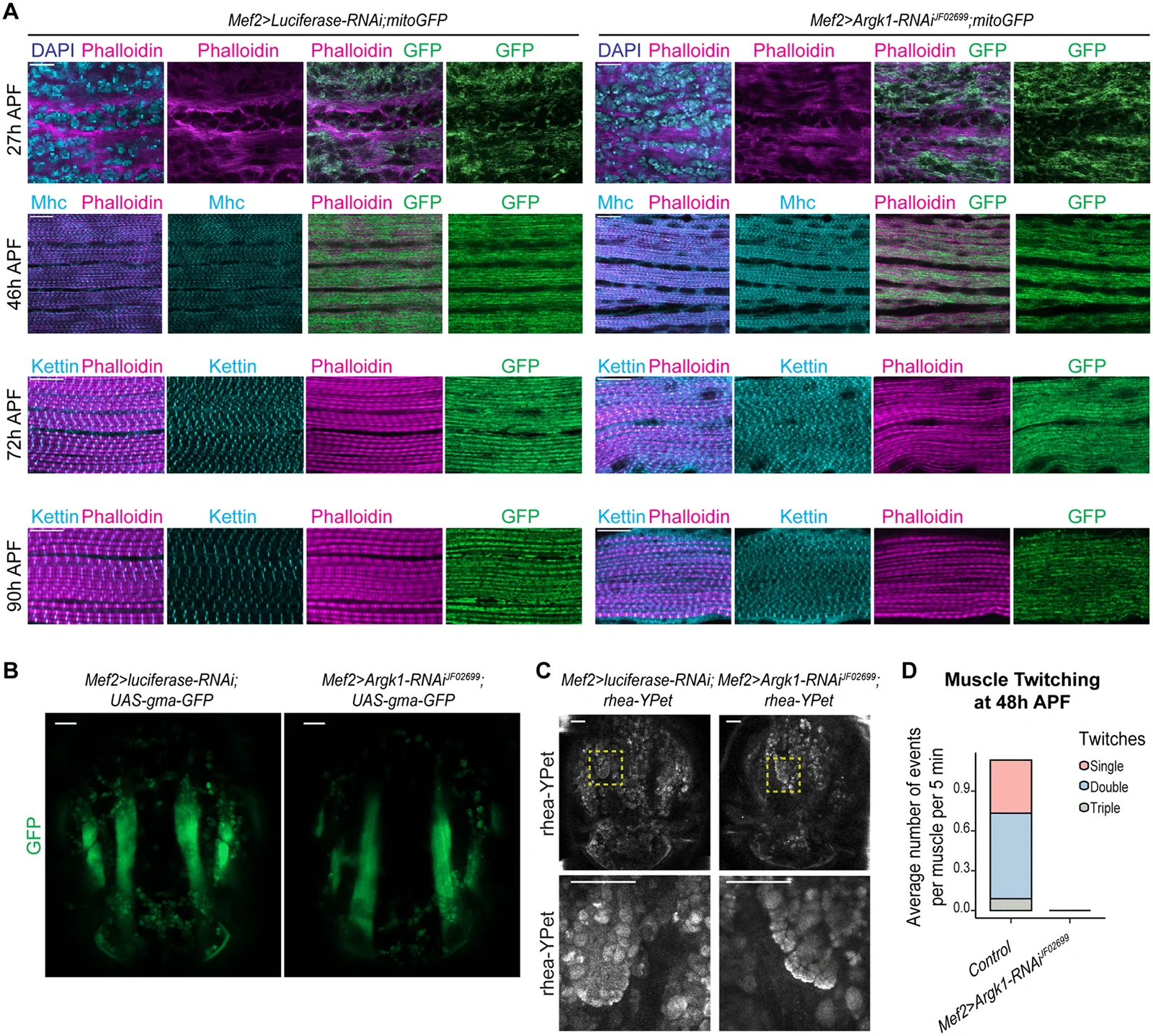
Argk1-depleted muscle completely failed to show spontaneous muscle twitching during myofibrillogenesis in developing IFM. (**A**) The structure of the IFM examined in *Mef2 > Argk1-RNAi*^*JF02699*^ and control *Mef2 > mCherry-RNAi* animals staged at 27, 46, 72, and 90h APF. Confocal single plane images of DLM-IFM in a sagittal view. Hemithorax sections stained with Phalloidin, DAPI, anti-Mhc, anti-Kettin and labeled with *UAS-mito.GFP*. (**B-C**) Confocal single plane of live imaging of developing flights muscles from *Mef2 > Argk1-RNAi*^*JF02699*^ and control *Mef2 > Luciferase-RNAi* pupa staged at 48h APF in a dorsal view. Anterior is to the top. (**B**) muscles are labeled with *UAS-gma-GFP*. (**C**) Muscle attachment site is labeled with *rhea-YPet*. The yellow-dashed box indicates magnified region for the recorded area in bottom panel. For live movies see S2 and S3 Movies. (**D**) Quantification of spontaneous contraction events per muscle per 5 min. Single, double and triple twitches were counted in developing flight muscles staged at 48h APF from 15 min live recordings from C. Stacked bars. N = 15–16 muscles/genotype. Scale is (**A**) 10 µm and (**B-C**) 50 µm. Genotypes are: (**A**) w; *UAS-mito.GFP*/ + ; *Mef2-GAL4*/*UAS-mCherry-RNAi*w; *UAS-mito.GFP*/ + ; *Mef2-GAL4*/*UAS-Argk1-RNAi*^*JF02699*^ (**B**) w; + ; *UAS-Luciferase-RNAi*/*Mef2-GAL4,* UAS-*gma-GFP*w; +/+; *UAS-Argk1-RNAi*^*JF02699*^/*Mef2-GAL4,* UAS-*gma-GFP* (**C**) w; *Mef2-GAL4*/ +  *UAS-Luciferase-RNAi*/*rhea-YPet*w; *Mef2-GAL4*/ + ; *UAS-Argk1-RNAi*^*JF02699*^/*rhea-YPet* (**D**) w; *Mef2-GAL4*/ + ; *UAS-Luciferase-RNAi*/*rhea-YPet* (control) w; *+* ;*rhea-YPet*/*rhea-YPet* (control) w; *Mef2-GAL4*/ + ; *UAS-Argk1-RNAi*^*JF02699*^/*rhea-YPet*.

Given that Argk-1-depleted flight muscles displayed a severe defect in the morphogenesis of myofibril and mitochondria, and in muscle growth (Fig 3B-3F), and that the peak of Argk1 expression in IFM is at 48h APF (Fig 1B), we examined Argk1-depleted muscle in further detail throughout IFM development [20,29]. The DLM-IFM were dissected at 27, 48, 72 and 90 h APF and stained with Phalloidin, antibodies against Mhc and kettin, while mitochondria were visualized with the *UAS-mito.GFP* reporter. At 27 h APF, the myotubes in *Mef2 > Argk1-RNAi* were indistinguishable from the control in *Mef2 > Luciferase-RNAi*. Normal filamentous network of mitochondria was detected in the myotubes (Fig 6A, top panel). At 46 h APF, Mhc organized at the A-bands was found at a regular pattern in *Mef2 > Argk1-RNAi*, thus suggesting that sarcomere assembly along with the formation of myofibrils initiated properly. At 72 h APF, the intercalation of mitochondria network between myofibrils was properly established in *Mef2 > Argk1-RNAi*. Yet, the Z-disc marker, Kettin, failed to properly assemble in these primitive sarcomeres in *Mef2 > Argk1-RNAi*, thus indicating compromised sarcomere maturation. Finally, at 90 h APF mitochondria completely failed to grow in *Mef2 > Argk1-RNAi* as we could not detect a visible increase in mitochondria volume, whereas large, elongated ellipsoids mitochondria were squeezed between myofibrils in the control *Mef2 > Luciferase-RNAi*. Moreover, thinner myofibrils and poorly defined sarcomeric structures were found in *Mef2 > Argk1-RNAi*, whereas fully mature sarcomere with regular, alternating stripes were present in the control (Fig 6A, bottom panel). Overall, our findings indicate an impairment in sarcomere maturation leading to myofibrillogenesis disruption in the Argk1-depleted muscles that precedes mitochondrial expansion in size.

Muscles undergo spontaneous contractions during development, which is important for the formation of cross-striated muscles [33]. In the IFM, the contractions can be first seen at the beginning of the sarcomere assembling in immature myofibrils at 30 h APF, with the peak at 48h APF, and at 72h APF the contractions cease [20]. To determine whether Argk1 knockdown affects these spontaneous contractions, we performed live imaging microscopy of IFM at 48h APF in intact pupa carrying transgenes: *Mef2 > gma-GFP* to label muscles (Fig 6B) and *rhea-YPet* label the muscle attachment sites for visualization of twitching events (Fig 6C, S2 Movie). In control pupa, about one twitching event consisting of either single, double or triple contractions was recorded every 5 minutes (Fig 6D). Strikingly, spontaneous contractions were completely absent in Argk1-depleted muscles. We did not detect a single twitching event in *Mef2 > Argk1-RNAi* animals (Fig 6D, S3 Movie). Significantly, the animals were alive during the recordings since very mild movements across all flight muscles were readily observed occasionally, and most animals further developed and reached pharate stage after the recordings.

Thus, Argk1-depleted muscles were unable to undergo spontaneous muscle contraction during pupal development, which is known to be an important process for proper sarcomere maturation in IFM [20,34] and cross-striation formation in abdominal muscles [33].

### Argk1 is required for myofibrillogenesis in the indirect flight muscle at late stages of development

**Fig 7:**
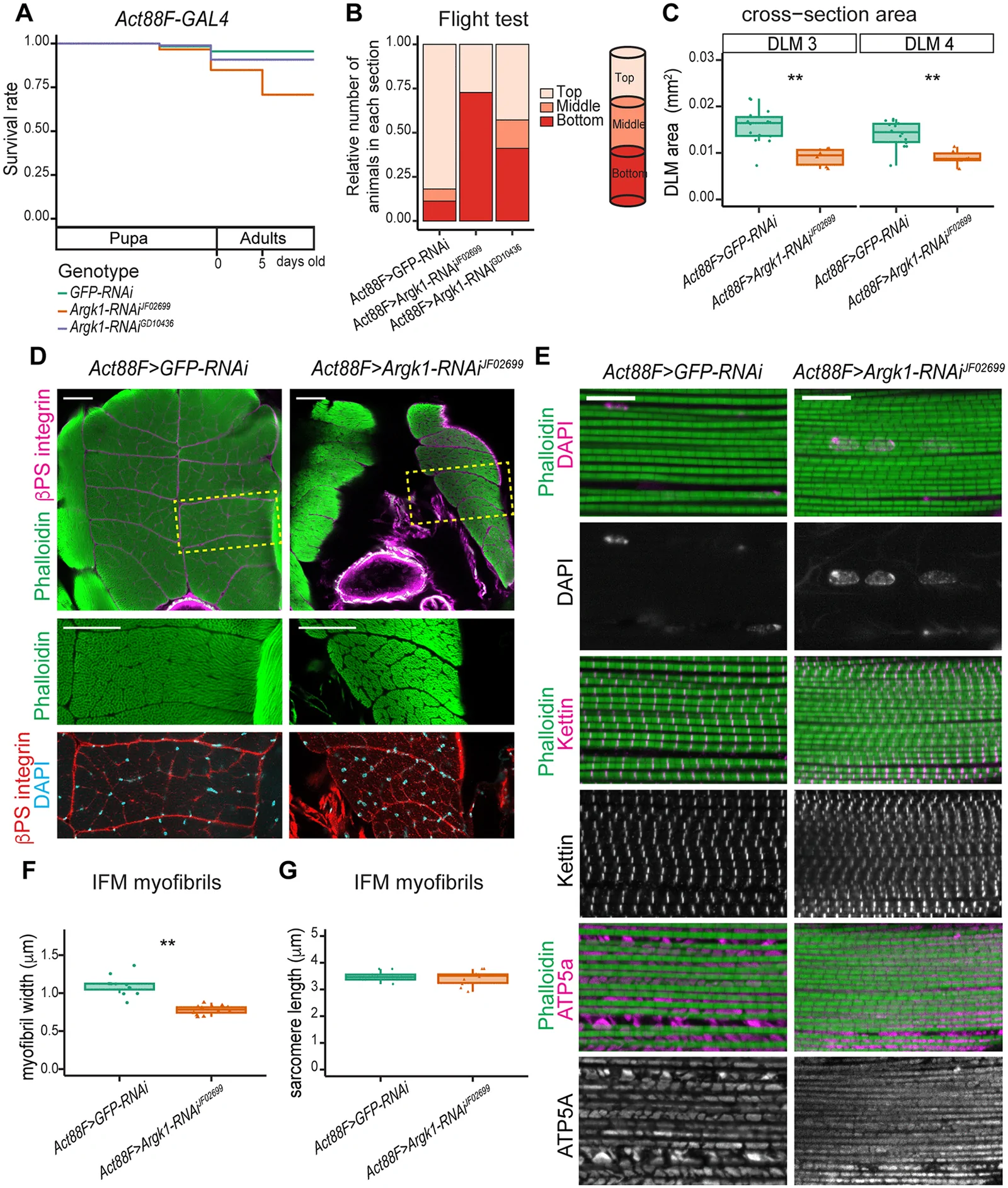
Argk1 knockdown with an indirect flight muscle-specific myofibrillogenesis confirms Argk1 requirement in sarcomere maturation. (**A**-**B**) Argk1 knockdown with *Act88F-GAL4* using two *Argk1-RNAi* lines, transgenes *JF02699* (stock 31959) and *GD10436* (stock 34036), compared to control *UAS-GFP-RNAi*. (A) Kaplan-Meier-like plot to illustrate survival rate of animals through late stages of development, only pupa and adult animals were quantified. Data represent mean of survival rate at each developmental stage scored. n = 90 flies per genotype. Experiment was repeated N = 3–4 times. (B) Flight ability scored by quantifying the percentage of flies landing on each section of the cylinder (top, middle and bottom). Stacked bars, n = 11–116 flies /genotype. The ability to flight of the animals that survived in **A** were assessed in **B**. Most males *Act88F>Argk1-RNAi*^*JF02699*^ died, and only 11 survived for the flight ability test. (**C-G**) The structure of the IFM examined in 1–2 days old *Act88F>Argk1-RNAi*^*JF02699*^ and control *Act88F>GFP-RNAi* animals. (**C**) Quantification of the cross-section area for muscle 3 and 4 from DLM-IFM per animal. N = 6–14 thoraces/genotype, Wilcoxon rank-sum test, Bonferroni-corrected for multiple comparison, ** p < 0.01. (**D**) Confocal single plane images of IFM in a transverse view DLM-IFM. Sections stained with Phalloidin (green), DAPI (cyan) and βPS-integrin (red and magenta). Yellow-dashed box indicates magnified area for muscle 4 from DLM-IFM (bottom panel). Dorsal up. (**E**) Confocal single plane images of DLM-IFM in a sagittal view. Hemithorax sections stained with Phalloidin (green), DAPI (magenta), anti-Kettin (magenta) and anti-ATP5A (magenta). (**F**) Quantification of myofibril width for the DLM-IFM per animal. N = 12 thoraces/genotype, Wilcoxon rank-sum test, ** p < 0.01. (**G**) Quantification of sarcomere length for the DLM-IFM per animal. N = 10 thoraces/genotype, Wilcoxon rank-sum test, p > 0.05. All box plot shows median (middle), interquartile range (box), 1.5x the interquartile range (whiskers), and individual data (points). Scale (**D**) 50 µm, and (**E**) 10 µm. Genotypes are: (**A-B**) *Act88F-GAL4*; *UAS-GFP-RNAi/+*; +*Act88F-GAL4*; + ; *UAS-Argk1-RNAi*^*JF02699*^*/+Act88F-GAL4*; + ; *UAS-Argk1-RNAi* ^*GD10436.34036*^*/+*(**C-G**) *Act88F-GAL4; +; UAS-mCherry-RNAi/+ Act88F-GAL4*; + ; *UAS-Argk1-RNAi*^*JF02699*^*/+*.

The results described above suggest that some of the defects in *Mef2 > Argk1-RNAi* muscle appear during mid pupal stages. To confirm that Argk1 is required at this point, we used the *Act88F-GAL4* driver that shows a more spatially and temporally restricted expression pattern in development. Unlike *Mef2-GAL4*, the *Act88F-GAL4* driver is expressed specifically in the IFM at the onset of myotube differentiation around 20 h APF to muscle maturation [35]. The knockdown of Argk1 under the control of *Act88F-GAL4* resulted in predominantly male lethality within five days post-eclosion (Fig 7A) and led to dysfunctional flight muscles as evidenced by a flight test (Fig 7B). Although the RNAi line *GD10436* did not result in lethality with *Act88F-GAL4*, the muscles of these animals were compromised, as revealed by the flight test (Fig 7A-7B). While the control animals landed at the top of the cylinder, almost half of *GD10436* and three quarters of *JF02699* flies failed to reach the top and landed at the bottom (Fig 7B), thus indicating that flight muscles were not fully functional.

These findings prompted us to examine the overall structure of the IFM. The transverse sections of thoraces from 5-days old adults stained with Phalloidin and anti-β-PS-integrin antibodies revealed a significant reduction in the IFM cross-sectional area, as quantified for muscle 3 and 4 from DLM-IFM (dashed yellow box), in *Act88F>Argk1-RNAi* ^*JF02699*^ compared to control flies (Fig 7C-7D). Moreover, sagittal sections stained with Phalloidin and anti-Kettin antibodies revealed reduced myofibril width in Argk1-depleted muscles albeit sarcomere length was normal (Fig 7E-7G). Moreover, sagittal sections stained with anti-ATP5A antibodies revealed slightly rounder mitochondria in Argk1-depleted muscles than in control. Thus, depletion of Argk1 in the developing IFM at the onset of myofibrillogenesis results in defects in myofibril and mitochondrial morphogenesis that, consequently, led to smaller and dysfunctional muscles.

## Discussion

The analysis of the transcriptional profiles of the wing disc-associated myoblasts by scRNA-seq offers a way to identify novel genes that are functionally important for early stages of flight muscle development. Indeed, many of the scRNA-seq derived markers for individual myoblast clusters were also isolated in a large-scale muscle-related RNAi screen [13]. In this work, we focused on *Argk1* that was highly expressed across several myoblast clusters and caused animal lethality upon its knockdown in the muscle [10]. Argk1 is well known for its importance in ATP buffering in cells with highly fluctuating energy demands, such as contracting muscles [36]. This is consistent with the broad view of the phosphagen system, which makes ATP available during bursts of high energy demand. Here, by examining the developing Argk1*-*depleted muscles, we uncovered a novel role for Argk1 in energy homeostasis. Argk1 is required to support spontaneous muscle contraction during flight muscle development that directly impacts myofibrillogenesis, sarcomere maturation, mitochondrial morphogenesis and muscle growth.

Argk1 belongs to the group of phosphagen kinases responsible for catalyzing the reversible transfer of a phosphate group between ATP and arginine, resulting in the formation of phosphoarginine. Since ATP is readily catalyzed by the phosphagen kinase during high energy turnover [37,38], phosphagens, such as phosphoarginine, serve as an on-demand reservoir of “high-energy phosphates”. The phosphagen kinases are important in proton buffering and in intracellular energy transport and play a dynamics role in the metabolic regulation of cells with high and variables rates of energy turnover, including muscles, neurons and spermatozoa [39]. In invertebrates, the activity of Arginine kinase has been associated with ATP regeneration and energy transport during burst of ATP demand in tissues with high level of energy metabolism [36,39,40]. The flight muscles is one of the highest metabolically demanding organs, as they undergo a 50–100-fold increase in oxygen consumption from rest to flight. Contracting flight muscles rely mainly on aerobic glycolysis and oxidative phosphorylation to generate ATP [41,42]. To maximize the efficiency of ATP delivery to the myosin ATPase for muscle contraction, the glycolytic enzymes are located within the sarcomere of the myofibrils [43,44]. Similarly, Argk1 is highly abundant in skeletal muscles [14,16,20,21] and is present within the sarcomere structures of the flight muscles that ensures localized ATP availability for muscle contraction [15]. Thus, Argk1 is commonly viewed as an important player in maintaining energy homeostasis during periods of high demand or burst of energy fluctuation to buffer the ATP levels. Our data are consistent with this established role of Argk1 since its depletion late in development leads to impaired muscle function in adults, which can be seen by the reduced performance of these animals in the flight test assay and by mild defects in sarcomere assembly and myofibrillogenesis. However, this view of Argk1 function appears to be incomplete, as our findings reveal that Argk1 is also required for cell growth during myoblast proliferation. This conclusion is supported by the reduced cell size of the wing disc-associated myoblasts and substantially reduced muscle size and severe defects in myofibrillogenesis in both direct and indirect flight muscles when Argk1 is depleted using early muscle specific GAL4 drivers. Accordingly, immunofluorescence and mRNA-seq show that Argk1 is highly expressed throughout flight muscle development [20], and therefore its expression is not limited to adults. Despite severe muscle defects, Argk1-depleted myoblasts properly turn on the transcriptional programs as they initiate and progress through early stages of differentiation without major disruption. We also did not find any alterations in cell cycle progression or cell division of proliferating myoblasts when Argk1 is depleted. However, Argk1 knockdown results in abnormal NAD + /NADH ratio and strong reduction of ATP levels, which is consistent with the role of Argk1 in maintaining energy homeostasis. Thus, our data support the idea that Argk1 is needed to sustain ATP levels and plays a direct role in flight muscle development.

During bursts of high energy demand, energy homeostasis in contracting muscles is effectively sustained because Argk1 catalyzes phosphotransfer from phosphoarginine to ADP practically instantaneously, thus regenerating ATP [37,38]. In vertebrates, creatine kinase, the functional analog of Argk1, shuttles high-energy phosphate in the form of phosphocreatine from mitochondria to sites of high energy consumption. This energetic shuttle is particularly efficient because phosphocreatine diffuses through the cytoplasm faster than ATP. Different creatine kinase isoforms with defined subcellular localization play a key role in defining intracellular route of the shuttle. The mitochondrial isoform, the M-type, that converts newly exported ATP into phosphocreatine, and the cytosolic isoform, the C-type, that regenerate local ATP from phosphocreatine at the site of highest demand [17]. Given the biochemical similarities between the phosphagen kinases, there is growing evidence supporting the role for Argk1 in buffering ATP to meet the high energy demand required for the contractile apparatus. Notably, the Argk1::GFP trap line largely showed mitochondrial distribution in the developing muscles. Based on the predicted splice isoforms generated by the transposon-mediated GFP insertion of MIMIC cassette, the reporter likely reflects the expression of only two out of seven Argk1 splice isoforms, *Argk1-RA*, and *Argk1-RE*. Concordantly, Argk1 is also found to be highly expressed in the muscle pioneer cells and larval body wall muscles and in mitochondria of motoneurons [22,23]. Given that Argk1 was previously reported to be localized to Z-bands in flight muscles [15], a likely explanation is that the isoform specifically localized within the sarcomere is not reported by the Argk1::GFP trap line. While many cytosolic and mitochondrial isoenzymes are encoded by different genes, there is growing evidence that Argk1 is the sole gene expressed in the developing flight muscles [20], showing spliced isoforms with differential subcellular localization [23,45].

Recent studies characterized spontaneous contractions of the IFM and abdominal muscles during their development [20,33,34]. Such muscle twitching events are important for muscle development since changes in the frequency of contractions affects cross-striation in abdominal muscles and sarcomere maturation in IFM. Interestingly, we found that Argk1-depleted muscles completely lack such twitching events and, concordantly, have abnormal myofibril and sarcomere structures. We suggest that Argk1 is needed to buffer ATP levels at the sites of the immature contractile sarcomeres to support spontaneous muscle twitching during flight muscle development, a function that is highly reminiscent to the established role of phosphagen kinases in adult muscle [39]. Thus, the main role of Argk1 is to make ATP readily available from the arginine phosphagen storage for the spontaneous contractions, which are likely mediated by ATPase-dependent myosin, in the developing IFM around 48 h APF. As spontaneous muscle twitching is one of the events required for proper muscle formation in IFM and abdominal muscles [20,33,34], we suggest that their absence may explain severe defects in sarcomere assembly in Argk1-depleted muscle. However, we cannot formally exclude the possibility that this could be the consequence of a prior defect.

Mitochondrial morphogenesis is tightly coordinated with myofibril morphogenesis and is mediated by mechanical tensions and kinesin-dependent microtubules [29,46], while mitochondrial fission and fusion are spatiotemporally regulated during muscle differentiation and growth [47]. Interestingly, the mitochondrial network is highly filamentous in Argk1-depleted muscle. Given that myofibrils mechanically constrain mitochondria into elongated shape in IFM [29], the defects in mitochondrial morphology observed in Argk1-depleted muscle could be the consequence of disrupted myofibrillogenesis. Such interpretation is in concordance with previous findings that highlight the importance in coordinating myofibrillogenesis with mitochondria morphogenesis.

In summary, our data suggest that the phosphagen kinase, Argk1, which is a known key player in energy homeostasis, is critical for spontaneous muscle twitching, an energetically demanding process that is required for proper myofibrillogenesis, mitochondrial morphogenesis and muscle growth. A transcriptional program that includes the OXPHOS and glycolytic-related genes is induced at around 30 h APF and peaks later in myofibrillogenesis [20,34,48]. We suggest that at the onset of myofibrillogenesis when ATP demand exceeds the capacity of glycolysis and oxidative phosphorylation to respond, Argk1 likely sustains local ATP levels by catalyzing the phosphotransfer from phosphoarginine to ADP at the sites of the immature contractile sarcomeres. Concomitantly, it regenerates phosphoarginine using newly synthesized ATP near the mitochondria. Thus, our work highlights a novel function for Argk1 in sustaining energy homeostasis during the spontaneous contractions taking place at the onset of myofibrillogenesis in IFM development.

## Methods

### Fly maintenance and stocks

Fly stocks were kept in vials with standard cornmeal food at 25°C. The *Mef2-GAL4* driver *P{GAL4-Mef2.R}3* (stock number 27390), *htl-GAL4* driver *P{GMR93H07-GAL4}attP2* (stock number 40669), the GFP-tagged stocks *P{PTT-GB}Argk1*^*CB03789*^ (stock number 51522), *PBac{Sp1-EGFP.S}VK00033* (stock number 38669) [49], *P{w[+mC]=UAS-mCherry.mito.OMM}3* (stock number 66533) and the UAS-RNAi lines from the TRIP collection: *UAS-Argk1-RNAi*^*JF02699*^ (*P{TRiP.JF02699}attP2*, stock number 31959), *UAS-Luciferase-RNAi* (*P{TRiP.JF01355}attP2*, stock number 31603) and *UAS-mCherry-RNAi* (*P{VALIUM20-mCherry}attP2*, stock number 35785) were obtained from the Bloomington Drosophila Stock Center (Bloomington, IN). The line *UAS-Argk1-RNAi*^*GD10436*^ was obtained from the library RNAi-GD (Construct ID GD10436, stock numbers 34036 and 34037) at the Vienna Drosophila Resource Center (Vienna, Austria). Most experiments used stock v34036. The line *Act88F-GAL4* [35], *1151-GAL4* [5] and *P{twi-LacZ}* were a gift from Richard. M. Cripps, the *htl-LexA* [30], *LexO-CD8-GFP*, and *LexO-nls-RFP* were from Sougata Roy, the *Him-CD8-GFP* F1068, *rhea-YPet* [20], and *UAS-gap-GFP* were from Maria Spletter and Frank Schnorrer, and E*(spl)m6-BFM-gap-GFP* [50] was from Krzysztof Jagla.

### Flight test

Flight tests were done as previously [51]. Briefly, 5 days-old males were collected into groups of 25 flies per vial, and recovered at 25 °C for 24 hours, followed by a one-hour acclimatation period at room temperature prior to the assay. Flies were flipped into a 2,000 mL cylinder lined with a piece of paper coated with mineral oil and immediately imaged. The distribution of the flies across three sections (top, middle and bottom) was automatically quantified by ImageJ. Then the number of flies landing in each section relative to the total was used to analyze data.

### Fly viability assay

The total number of pupae, pharate adults, and adults were scored relative to the total as previously done [52]. Pupal developmental stages were determined following staging criteria as in [53].

### ATP determination

The ATP assay was performed following manufacturer’s guidelines and adapted from [51,54]. A total of 2 females and 2 males staged at 90 h APF (pharate) were dissected out of the pupa case and homogenized in 200 µl of extraction buffer (6 M guanidine-HCl, 100 mM Tris (pH 7.5), and 4mM EDTA (pH 8)). An aliquot was saved to measure protein concentration by the Bradford assay (Bio-Rad 500–0006). Samples were immediately boiled in a heat block for 5 min, and centrifuged at 14,500 rpm for 5 min at 4°C. The supernatant was transferred to a fresh Eppendorf tube. The content of ATP was measured using the ATP determination kit (Molecular probes #A22066) with an ATP standard curve in a white opaque 96-well plate (Corning). The luminescence was measured in the Synergy H1 microplate reader (Biotek). The ATP level was normalized to total protein concentration. A total of 4 or 5 samples per group were measured at once.

### NAD+ and NADH measurement

The Amplite fluorimetric NAD + /NADH ratio assay kit was performed following manufacturer’s guidelines (red fluorescence, AAT Bioquest 15263). A total of 3 females and 2 males staged at 90 h APF (pharate) were dissected out of the pupa case and homogenized in 150 µl of lysis buffer. Samples were immediately centrifuged at 3,000 g for 10 min at 4°C. The supernatant was transferred to a fresh Eppendorf tube and incubated at 37°C for 15 min. Samples were loaded into a solid black 96-well plate (Corning) and incubated either with NADH Extraction Solution then neutralized by NAD Extraction Solution or with NAD Extraction Solution then neutralized by NADH Extraction Solution. The NAD/NADH working solution was added into each well and incubated for 15 min at 25 °C. The fluorescence was measured at Ex/Em = 540/590 nm in the Synergy H1 microplate reader (Biotek). The levels of NAD+ and NADH in each sample was determined using the NADH standard curve, and the NAD+ values were normalized to the NADH values for each sample. A total of 5 samples per group were measured at once.

### Immunostaining and confocal imaging

For wing discs, third instar larvae were dissected in phosphate-buffered saline (PBS) at pH 7.4 and immediately fixed with 4% formaldehyde for 30 min. Incubation was reduced to 15 min in ice when anti-Ct antibody was used. Then, tissue was permeabilized twice in 0.3% Triton X-100 in PBS 1x (PBT) for 10 min each and blocked in 10% normal donkey serum (NDS) in 0.1% PBT for 1 h. Following primary antibody incubation tissue was washed for 5 min three times with 0.1% PBT, then incubated with secondary antibodies and fluorescent dyes in blocking buffer for 1 h covered from the light. Finally, samples were washed five times with 0.1% PBT for 5 min each.

For developing flight muscles, pupae were staged at 20 h, 24 h, 30 h and 45 h APF and dissected as in [25]. Briefly, tissue was fixed for 15 min, permeabilized three times for 20 min each and blocked for 2 h. After incubation of the primary antibody, tissue was washed four times for 25 min each, incubated with secondary antibody for 2 h in blocking buffer, and washed four times for 15 min each.

For transverse sections of adult and pharate flight muscles, whole animals were snap-frozen in liquid nitrogen for two minutes. Thoraces were cut twice in a transversal plane with a razor and fixed for 2 h in 4% formaldehyde in relaxing buffer (20 mM phosphate buffer pH 7.0, 5 mM MgCl2, 5 mM EGTA, 0.1% Triton-X, 5 mM ATP). For the sagittal sections of 72 h, 90 h APF and adult flight muscles, animals were incubated in relaxing buffer for 15 min, fixed in 4% formaldehyde in relaxing buffer for 30 min, cut through the appropriate sagittal plane with a Sharpoint 22.5° Stab Knife (ID#72–2201) and fixed again for 15 min. Tissues were permeabilized for 15 min four times with 0.3% PBT for IFM sections and 0.5% PBT for DFM sections, and blocked for 2 h in 2% bovine serum albumin (BSA) 0.3% PBT. After primary antibody incubation, tissues were washed four times with PBT for 15 min. Secondary antibody was incubated for 2 h in 10% NDS 0.3% PBT for IFM sections, and 10% NDS 0.5% PBT for DFM sections. Finally, sections were washed for 10 min four times each.

All primary antibodies were incubated overnight at 4°C in blocking buffer. Primary and secondary antibodies and fluorescent dyes are listed in S2 Table. Samples were stored in 0.5% propyl gallate glycerol at 4°C until mounted. All steps were carried out at room temperature with gentle rocking unless noted.

Fluorescent images were acquired using Laser Scanning Microscope 700 (Zeiss LSM700, Observer.Z1) using x20/0.8, x40/1.2 and x100/1.45 objectives and Laser Scanning Microscope 900 (Zeiss LSM900 Airyscan 2, motorized Axio Observer 7) using x20/0.8, x40/1.2W, x63/1.4 oil. Pinhole was set at 1 AU, laser and gain were not adjusted within experiments. High resolution images of mitochondrial structure in Figs 1, S2G, and 6 were acquired using the 63x objective configured with the Airyscan2 detector (32-channel GaAsP detector array) and set up with the Airyscan super-resolution (SR) mode. Raw data were processed using ZEISS ZEN 3.8 Software Suite using Airyscan processing algorithm. Images were processed using ImageJ and Photoshop (Adobe Systems). Only representative images are shown.

### Live imaging and analysis

Live imaging of the developing flight muscles were done as previously [20,55] with the following modifications. Animals were staged at 48 h APF, and a small window was surgically opened in the pupa case over the dorsal thorax. Pupae were gently positioned in a Vaseline-coated groove slide and mounted with 50% glycerol for immediate visualization. Imaging was done using a Zeiss LSM900 microscope. Temperature was maintained at 25°C with controlled humidity using a stage-top live-cell incubation system, equipped with a heating insert and the Definite Focus.3. Time-lapse recordings were acquired at 0.4 s intervals for a total duration of 15 min using x20/0.8. Following the imaging session, pupae were kept in the incubator at 25°C to monitor for successful development, thus ensuring animals were viables during recording. Videos were created at 20 fps using ImageJ and randomized for muscle twitching quantification. The number of single, double and triple muscle twitches were scored per muscle. Only one muscle was quantified per video.

### Tissue dissociation into a single-cell suspension

Wing discs from wandering third instar larvae were dissected for 60 min. Discs were harvested in chilled HBSS + 0.1% BSA. Tissue was dissociated for 15 min in 100–200 μl dissociation buffer (2 mg/ml collagenase A (Roche 11088793001), 0.8 U/ml Dispase II (Roche 04942078001), 2 mM CaCl_2_ in HBSS) in an Eppendorf Thermo mixer C set at 30°C, 500 rpm. Cells were washed two times with resuspension buffer by centrifugating at 800 g for 5 min at 4°C, and filtered. Cell viability and concentration was assessed by staining cells with Trypan blue and counting cells using a hemocytometer. Only samples showing cell viability over 90% were used for the downstream applications scRNA-seq and FACS. For scRNAseq application, the resuspension buffer was 0.5% BSA PBS 1x and two consecutive cell strainers were used: a Bel-Art Flowmi 40 μm and a mini-strainer 10 μm (43-10010-40). For FACS application the resuspension buffer was 0.5% BSA 2 mM EDTA PBS 1x and the cell strainer used was a Bel-Art Flowmi 40 μm. Samples and media were stored on ice unless otherwise noted.

### Fluorescence-Activated Cell Sorting (FACS)

Flow cytometric analysis was followed as in [56,57]. After tissue dissociation, cells were stained with Hoechst 33342 1 μg/mL, and propidium iodide 5 μg/mL in the resuspension buffer for 10 min in a covered Eppendorf Thermo mixer C set at 25°C, 300 rpm. Then, cells were immediately transferred to a 5 mL polystyrene round bottom tube and sorted using the CytoFLEX S (Beckman Coulter). The software CytExpert was used to set up the run. To remove debris, clumps and damaged cells, and to ensure the analysis of individual cells, five consecutive gates were applied using (1) Forward scatter (FSC) area vs side scatter (SSC) area,(2) FSC height vs FSC area, (3) SSC height vs SSC width, (4) channel PB450 height vs channel PB450 area, and (5) channel PB450 height vs channel PI height. Then, the myoblasts were selected using FITC channel. Over 7,000 events that were positive for both GFP and Hoechst were selected for downstream analysis. Data were analyzed and visualized using Flow Jo v10. DNA content was measured using Hoechst and the cell cycle phases G1, S and G2 were calculated using Dean-Jett-Fox model. The relative size was estimated based on the FSC height for cells in G1.

### Library preparation and sequencing for scRNAseq

Chromium Single-Cell 3’ Reagent kits v3 for first experiment and Chromium Next GEM Single-Cell 3’ Reagent kits v3.1 for second experiment were used according to the manufacturer’s protocol (10x Genomics, CG000183RevA and CG000315 Rev C, respectively). Around 10,000 cells were targeted per sample in the Chromium Controller for the first experiment and around 20,000 cells in the Chromium X for the second experiment. Libraries were single-indexed for the first experiment, while dual indexed for the second experiment. Quality of both amplified cDNA and libraries was assessed using Agilent TapeStation 4200 instrument. The libraries were pooled and diluted for sequencing onto HiSeq, NovaSeq 6000 and NovaSeq X Plus instruments (Illumina). Sequencing was done at the Single Cell Sequencing Pilot at the University of Illinois at Chicago, University of Illinois at Chicago Sequencing Core (UICSQC), the DNA services at University of Illinois at Urbana-Champaign, and Novogene. Sequencing saturation was over 46% with over 63,000 reads per cell.

### scRNA-seq data analysis

First, sequence reads were processed through IndexHopping (v1.1.0). The alignment, filtering, barcode counting and UMI counting was done using Cell Ranger (v7.1.0) with the command count and default settings. The reference genome was *Drosophila melanogaster* BDGP6.32 (Ensembl version 109). Downstream analysis was done in R (v4.4.1) using standard workflow from Seurat package (v5.3.0) [32]. For pre-processing, the low-quality cells were removed from the dataset. We used min.cells = 5, along with 1,000 as low and 5,000 as top cut-off for gene number per cell, 10% for mitochondrial content, and 55,000 UMI per cell. Doublets and multiplets were removed from each sample using DoubletFinder (v2.0.4). Epithelial cells, tracheal cells and myoblasts were identified based on the expression of the respective markers: *Fasciclin 3* (*Fas3*) and *grainy head* (*grh*) for epithelia, *trachealess* (*trh*) for trachea, and *Zn finger domain 1* (*zfh1*), *Holes in muscles* (*Him*) and *Secreted protein, acidic, cysteine-rich* (*SPARC*) for myoblast. The myoblasts were subset for downstream analysis. Cluster showing high expression of epithelial markers and mitochondrial encoded genes were removed as contaminant clusters. The barcodes of cell used in downstream analysis are listed in S1 Data. Normalization was done using SCTransform v2 algorithm with the glmGamPoi package to correct for technical factors. Cell cycle phase scores were calculated using the function CellCycleScoring based on the markers for S and G2/M phase genes [58]. The cell-cycle score was regressed along with the lncRNA:roX genes and mitochondrial content to minimize their impact in clustering. Note that the clusters that did not show even representation across the replicates were removed from the dataset as potential sample bias. Dimensional reduction was done through principal component analysis using the top 3,000 high variable genes. Cell clustering was performed by the non-linear dimensional reduction UMAP using 24 principal components and Leiden algorithm. The optimal resolution was determined using the ClustreTree package across the range 0.5 - 1.7. Clusters with unique expression markers were selected at resolution 1.2, and some of the small clusters missing unique markers were merged back. The biomarkers for each cluster were determined by differential expression analysis using the FindAllMarkers function with Wilcoxon Rank Sum test. Clusters were annotated based on the cluster-specific biomarkers published in the reference atlas for wing-disc associated myoblasts [10]. Differential gene expression analysis between Argk1-depleted and control myoblasts was determined using a pseudobulk approach. Raw counts were aggregated per cell cluster and sample. Differential expression was tested using FindMarkers function with DESeq2 test. Genes with adjusted p-value < 0.1 (Bonferroni correction) were considered as significant changes.

### Quantification and statistical analysis

Myoblast count: Confocal images of larval wing discs were taken with LSM700 40x/1.2 and processed with Fiji. The region of interest (ROI) in the myoblasts was set in a focal plane with consistent expression of *htl > nls-RFP*. Total number of RFP positive cells and number of DAPI positive cells were manually counted in each ROI.

Myoblast layer depth: Notum of wing imaginal discs was imaged with Zeiss LSM700 using 20x/0.8. The z-stacks were randomized, and the XZ orthogonal sections were generated using Fiji. The length of myoblast layer was measured across the GFP positive area, and the total length of the proper discs was measured across the total tissue area (from basal to apical). A total of three measurements per image were averaged to minimize technical variation from disc folding and mounting.

Mitotic index: Confocal images of larval wing discs stained with the mitotic marker pH3 and DAPI were taken with LSM700 20x/0.8 and processed with Fiji. The ROI in the myoblasts were set in a focal plane with consistent expression of *twi-lacZ* reporter. Total number of pH3 positive cells per ROI was manually counted and total number of DAPI per ROI was automatically counted using Analyze Particle function. The mitotic index was determined as number of pH3 cells relative to total (DAPI) in each ROI [59].

Cross-sectional area: Both muscles 3 and 4 from DLM-IFM in a transverse section were measured using Fiji. The area of the left and right muscles was averaged per animal [52].

Myofibril width and sarcomere length: Confocal images of the DLM-IFM sagittal sections taken with LSM700 100x/1.45 were split and phalloidin staining was processed using the Fiji plug-in MyofibrilJ [20]. The sarcomere length (indicated as repeatGlobal) and myofibril width (indicated as thicknessGlobal) were determined per image.

Mitochondria shape: Confocal images of the DLM-IFM sagittal section taken with LSM700 100x/1.45 were randomized. Only the ATP5A channel was used for blind quantification. Hemithorax was scored as either ellipsoid or filamentous/round regarding the mitochondrial network [52].

Statistical analysis was done using Rstudio (v4.4.3). Details regarding data presentation and statistical analysis were included in legends to Figures for each experiment.

## Supporting information

S1 Fig Argk1 is expressed throughout flight muscle development. Related to Fig 1. The expression of Argk1 (Argk1::GFP) was examined in flight muscle development using the GFP trap line *Argk1*^CB03789^. (**A**) Confocal single plane image of third instar larval wing discs stained with anti-Ct (cyan, magenta) and *mCherry.mito.OMM* (magenta). (**B**) Confocal single plane image of forming DLM-IFM sagittal view at 90 h stained with Phalloidin (red and magenta) and DAPI. Genotypes are: (A) *w*; + ; *P{PTT-GB}Argk1*^*CB03789*^/ *Mef2 > mCherry.mito.OMM* (B*) w*; + ; *P{PTT-GB}Argk1*^*CB03789*^/+ Scale (A) 50 µm and (B) 20 µm.
(TIF)

S2 Fig Argk1 knockdown driven by *Mef2-GAL4* results in severe defects in both IFM and DFM. Related to Fig 3. The structure of the IFM examined in *Mef2 > Argk1-RNAi*^*GD10436*^ and control *Mef2 > mCherry-RNAi* animals at 1 or 2-days-old. (**A**) Confocal single plane images of IFM in a transverse view of the DLM-IFM. Sections stained with Phalloidin (green), DAPI (cyan) and βPS-integrin (red and magenta). Yellow-dashed box indicates magnified area for muscle 4 from DLM-IFM (bottom panel). Dorsal up. (**B**) Quantification of the cross-section area for muscle 3 and 4 from DLM-IFM per animal. N = 6–7 thoraces/genotype, Wilcoxon rank-sum test, Bonferroni-corrected for multiple comparison, * p < 0.05. (**C**) Confocal single plane images of DFM (muscle 53, left panel) and IFM (dorsal longitudinal muscles, right panel) in a sagittal view. Hemithorax sections stained with Phalloidin (green), DAPI (magenta), anti-Kettin (magenta) and anti-ATP5A (magenta). (**D**) Quantification of myofibril width for the DLM-IFM per animal. N = 12–14 thoraces/genotype, Wilcoxon rank-sum test, ** p < 0.01. (**E**) Quantification of sarcomere length for the DLM-IFM per animal. N = 8–12 thoraces/genotype, Wilcoxon rank-sum test, ** p < 0.01. (**F**) Blind quantification of the number of hemi-thoraces displaying either proper morphology of mitochondria (ellipsoid) or altered mitochondrial shape (round) from DLM-IFM. Stacked bars. N = 15–18 hemithoraces/genotype. N = 2 independent experiments. (**G**) Confocal single plane images of DLM-IFM in a sagittal view. Hemithorax sections stained with Phalloidin (magenta), anti-Kettin (cyan) and labeled with *UAS-mito.GFP* (green). All box plot shows median (middle), interquartile range (box), 1.5x the interquartile range (whiskers), and individual data (points). Scale (A) 50 µm, and (C, G) 10 µm. Genotypes are: (**A-F**) w; + ; *Mef2-GAL4/UAS-mCherry-RNAi* w; + ; *Mef2-GAL4/UAS-Argk1-RNAi* ^*GD10436*^ (**G**) w; *UAS-mito.GFP/ +* ; *Mef2-GAL4/UAS-Luciferase-RNAi* w; *UAS-mito.GFP/ +* ; *Mef2-GAL4/UAS-Argk1-RNAi* ^*GD10436*^
(TIF)

S3 Fig Argk1 knockdown does not alter the overall identity of the undifferentiated and differentiating myoblasts. Related to Fig 5. (A) Hierarchical heatmap showing the expression levels of 165 genes listed as the top 40 markers per cluster (adjp<0.05, log2FC>0.8, pct1 > 0.4). Hierarchical clustering by k-means to group the genes into 6 groups based on similarity. Color intensity shows the average expression level normalized and scaled. (B) Two-dimensional UMAP representation of both the combined *Mef2 > Argk1-* RNAi^JF02699^ (5,681 myoblasts) and *Mef2 > mCherry-RNAi* (5,808 myoblasts) from third instar larval wing discs datasets, colored by cell cluster and split by replicate. (C) Total number of cells per cluster per sample relative to the total number of myoblasts per sample for the combined datasets *Mef2 > Argk1-* RNAi^JF02699^ and *Mef2 > mCherry-RNAi* across four samples. Genotypes are: w; + ; *Mef2-GAL4/UAS-mCherry-RNAi*w; + ; *Mef2-GAL4/UAS-Argk1-RNAi*^*JF02699*^
(TIF)

S4 Fig The loss of Argk1 in myoblasts shows only few genes differentially expressed. Related to Fig 5. Volcano plots showing differentially expressed genes between *Mef2 > Argk1-* RNAi^JF02699^ and *Mef2 > mCherry-RNAi* myoblasts across (**A**) individual clusters and (**B**) all clusters grouped together. Differential expression was determined by pseudobulk analysis (FindMarkers, DE-Seq2 test), dashed line represents adj p < 0.1. Volcano plots illustrate fold change (Log2) and adj p-value (-Log10). Genes showing significant changes are shown in red, the top 10 most significant genes per panel are labeled. Genotypes are: w; + ; *Mef2-GAL4/UAS-mCherry-RNAi*w; + ; *Mef2-GAL4/UAS-Argk1-RNAi*^*JF02699*^
(TIF)

S1 Movie Confocal z-stack image sequence of *Argk1*^CB03789^ in third instar larval wing discs displayed as a movie. Related to Fig 1. Expression of *Argk1*^CB03789^ in third instar larval wing disc-associated myoblasts. Movie created at 24 frames per second. Each frame corresponds to a discrete z-position taken at a 1.09 μm interval (z1 = 0, z2 = 1.09, z3 = 2.18, z4 = 3.27, z5 = 4.36, z6 = 5.45, z7 = 6.54, z8 = 7.63, z9 = 8.72 and z10 = 9.81) Genotype is *w*; + ; *P{PTT-GB}Argk1*^*CB03789*^/+Scale 50 µm.
(AVI)

S2 Movie Live imaging of *Mef2 > Luciferase-RNAi* developing flights muscles. Related to Fig 6. Animal staged at 48h APF recorded in a dorsal view. Anterior to top. Muscle attachment site labeled with rhea-YPet. Movie created at 20 frames per second. Genotype is *w; Mef2-GAL4/ + ; rhea-YPet/UAS-Luciferase-RNAi*.
(AVI)

S3 Movie Live imaging of *Mef2 > Argk1-RNAi*^*JF02699*^ developing flights muscles. Related to Fig 6. Animal staged at 48h APF recorded in a dorsal view. Anterior to top. Muscle attachment site labeled with *rhea-YPet*. Movie created at 20 frames per second. Genotype is *w; Mef2-GAL4/ + ; rhea-YPet/UAS-Argk1-RNAi*^*JF02699*^
(AVI)

S1 Table Cell count per cluster and sample. (XLSX)

S2 Table List of antibodies and fluorescent dyes used for immunofluorescence. (XLSX)

S1 Data List of cell barcodes used after applying quality control filters and selecting the myoblasts. Related to Fig 5.
(XLSX)

S2 Data Markers for each cluster of the combined dataset *Mef2 > mCherry-RNAi* and *Mef2 > Argk-RNAi* myoblasts. Related to Fig 5. List of all differentially expressed genes across all clusters. Only positive markers with threshold set p_val_adj < 0.05.
(XLSX)

S3 Data Average expression of all genes across all clusters of the combined dataset *Mef2 > mCherry-RNAi* and *Mef2 > Argk-RNAi* myoblasts. Related to Fig 5. List of average gene expression across all clusters (SCT assay).
(XLSX)

S4 Data Top biomarkers per cluster grouped into 6 groups by k-means of the combined dataset *Mef2 > mCherry-RNAi* and *Mef2 > Argk-RNAi* myoblasts. Related to S4 Fig. The top unique 40 markers per cluster (165 genes, adjp<0.05, log2FC>0.8, pct1 > 0.4) clustered by k-means to group genes based on similarity.
(XLSX)

S5 Data Aggregate expression across genotypes and across samples of the combined dataset *Mef2 > mCherry-RNAi* and *Mef2 > Argk-RNAi* myoblasts. Related to Fig 5. Aggregate expression across the two genotypes and across all four samples.
(XLSX)

S6 Data Differential expression using pseudobulk (DESeq2) between *Mef2 > mCherry-RNAi* and *Mef2 > Argk-RNAi* myoblasts. Related to S4 Fig. List of differentially expressed genes between *Mef2 > mCherry-RNAi* and *Mef2 > Argk1-RNAi* (DE-Seq2, p < 0.05) for each cluster and all clusters together. Threshold set to adj_p < 0.1 to assign significant changes in red.
(XLSX)

S7 Data Numerical data. Related to Figs 2, 3, 4, 6, 7, S2. Data underlying graphs organized by Figure panels
(XLSX)
