# Supplementary material for: Arginine Kinase 1 supports energy homeostasis in Drosophila flight muscle development
Source: PLoS Genet. 2026 Sep 17;22(9):e1012304. doi: 10.1371/journal.pgen.1012304 (PMC13585230; doi:10.1371/journal.pgen.1012304)
Supplement: Supplementary file 1 [file pgen.1012304.s017.docx]

**Reviewers' comments:**

**Reviewer #1:** *Zappia and colleagues, analyze the role the Drosophila arginine kinase 1 (Argk1) plays in flight muscle development. The authors’ logic to study Argk1 in this developmental process is that it’s muscle-specific knockdown results in pupal lethality and thus it may have an important function during muscle development. While not untrue, it is a weak basis for its specific study since 1. numerous RNAi screens over many years, some dating more than 15 years, using the same strategy have revealed very many genes with lethal pupal phenotypes and 2. Drosophila flight muscle is not required for viability.
A more logical and valid reason for its study is the role Argk1 plays in energy homeostasis : Argk1 is one of four Drosophila arginine kinases that catalyze the transfer of phosphate from ATP to arginine, yielding phosphoarginine and ADP. They act as 'phosphagen kinases' in invertebrates, playing a key role as ATP buffering systems in cells that display high and variable rates of ATP turnover. As flight muscle is the most energy demanding tissue in Drosophila, the role Argk1 plays is thus potentially of interest.

While the experiments are by-and-large correct and well-performed, I feel the choice of experiments performed to study energy homeostasis are of little use. For example, a very complicated scRNA-seq was performed (Figure 6), whose conclusion was that Argk1 doesn’t generally affect muscle precursor identity. The authors have previously studied transcription factor function in flight muscle development, where scRNA-seq is an appropriate study. It is of little surprise, to this reviewer, that the identity of muscle precursors don’t change when one knocks down expression of a cytoplasmic arginine kinase. A bulk (or better single-cell) metabolomics study would perhaps have been more appropriate for studying the effects of knockdown a such an enzyme?*

**Response:** We thank the reviewer for the constructive comments on how to improve the manuscript. We agree with the reviewer that Argk1 is one of many genes with pupal lethal phenotype that were isolated in several RNAi muscle screens. However, this is not the only reason why we chose to study Argk1. Argk1 is a marker that we identified in single cell atlas that was prominently expressed across several cell clusters (EMBO Rep. 2020;21(10):e49555. doi: 10.15252/embr.201949555). Given that several genes with known roles in muscle development are among the scRNA-seq identified markers, we asked whether Argk1 may also have a previously unrecognized role in muscle development. We consider this to be an important conceptual point with potential implications beyond muscle development as such approach provides a framework to mine other single cell atlases to identify novel genes. Indeed, this approach led to the identification of *Amalgam* as an important gene in glial cell development (J Cell Sci. 2020;133(19):jcs250837. doi: 10.1242/jcs.250837).

We appreciate that the reviewer noted that the experiments are correct and well-performed. The reviewer asked why we performed scRNA-seq since Argk1 is a metabolic enzyme. We agree with the reviewer that Argk1 is unlikely to infer direct transcriptional effects. The reason we employed scRNA-seq was to determine whether Argk1-depleted cells progress normally through early stages of differentiation. scRNA-seq provides a highly detailed view of the process and at a much higher resolution than looking at a few markers by immunofluorescence. This allowed us to unambiguously conclude that early stages of differentiation were not severely impaired in Argk1-depleted myoblasts. We hope that the reviewer would agree.

New data were added to the manuscript in Figure 1 and 6. Text and conclusions were revised accordingly.

*Since the scRNA-seq approach did not change the identity of the muscle precursors, they analyzed their single cell data as pseudo-bulk RNA-seq for differential gene expression analysis. Here, a very simple an inexpensive bulk RNA-seq would perhaps have yielded higher quality differential gene expression data (or not) permitting a p-value cut off much more stringent than 0.05 (why was p-adj not used?). Nevertheless, some genes were found as differentially expressed although whether upregulated or downregulated was not specified in the gene ontology enrichment analysis which hinted at defects in energy homeostasis. However, while the link is quite weak, metabolomic tests, limited to simple NAD+/NADH ratio and global ATP level tests demonstrated a reduction in both upon Argk1 knockdown. Again, why Argk1 knockdown would lead to the transcriptional up/down regulation of oxidoreductase enzymes is unclear and not discussed. Do knockdown of these oxidoreductase enzymes phenocopy the muscle phenotypes observed with that of Argk1?*

**Response:** We agree with the reviewer that the statistical power of our pseudobulk analysis is limited compared to a dedicated bulk RNA-seq given the shallow sequencing inherent to the scRNA-seq and the small number of replicates due to the high cost. However, bulk approaches might not be as informative given the presence of normal epithelial cells in each sample that will mask the effects of the loss of Argk1 in myoblasts. Nevertheless, we understand the reviewer’s point about the low p-values in GOBP enrichment analysis. Therefore, we applied the adj-p value, which is more stringent than p-value, and now present data as volcano plots showing up-regulated and down-regulated genes in Argk-depleted myoblasts compared to control. The data are shown in Figure S4, Data S6, and are described on page 19. Given the much lower number of differentially expressed genes when adj-p value was used, we removed the GOBP enrichment analysis, from the manuscript. Our conclusions were adjusted accordingly in the title, abstract, results (page 19), and discussion (pages 23,25,27).

*The authors then finally link the phenotypes observed upon Argk1 knockdown to mitochondrial network abnormalities to conclude it as an essential enzyme for energy homeostasis during muscle development. While it has been previously shown by Avellaneda and colleagues (doi: 10.1038/s41467-021-22058-7 ) that muscle and mitochondrial development are intricately intertwined, by Zappia and Frolov that factors controlling both myogenesis and mitochondrial biogenesis can affect both muscle and mitochondria morphologically (doi 10.1038/ncomms10509 ), the study by Poliacikova and colleagues (doi 10.1038/s41467-023-38986-5 ) shows that one can affect/eliminate only mitochondrial energy function, without affecting muscle development. Thus, it is extremely difficult to correlate that disruption to energy homeostasis is the reason for the observed impaired myogenesis. Furtheremore, two different ArgK1 isozymes have been reported in Drosophila (Munneke and Collier doi 10.1007/bf00555494 ): a smaller cytoplasmic form that locates at the Z line of myofibrils of IFM and a bigger mitochondrial form that locates in the mitochondrion (Lang et al doi 10.1007/bf00711796 ). Since the difference is in a longer N-terminal sequence, both forms will be affected by the RNAi. Have the authors considered that the muscle and mitochondrial phenotypes simply reflect two distinct functions for ArgK1 – one in the mitochondria and the other at the Z-line in the developing myofiber?*

**Response:** The reviewer makes an important point that Argk1 gene encodes several isoforms. Based on the genomic insertion site, the GFP trap line that we use to assess the expression of Argk1 tags GFP to the long isoform of Argk1 near the N-terminus (Figure 1C). New data presented in Figure 1 show Argk1::GFP localization to mitochondria in the developing flight muscles as well as in fully mature flight muscles (pages 9-10). This confirms that the long Argk 1 isoforms are localized to the mitochondria and is consistent with the paper by Munneke and Collier that the reviewer cited. We included a discussion of how different isoforms encoded by the Argk1 gene can have different localization and noted that the GFP line likely tags the isoform with the mitochondrial localization on pages 25-26.

*As energy homeostasis is the major interesting aspect of the study, a more in depth metabolomics study is required and/or RNAi of other enzymes required for energy production to try to understand how energy homeostasis is required for myogenesis. At a bare minimum, quantification of phosphorylarginine (the product of reversible transfer of a phosphate between ATP and arginine that is catalyzed by ArgK1) should be performed.*

**Response:** We agree that the metabolomic analysis will be informative, however, we believe that this is beyond the scope of this paper. The conclusions regarding the role of Argk1 in energy homeostasis during muscle development have been adjusted according to the data presented in the manuscript (title, abstract, introduction pages 7-8, result page 14 and discussion pages 23,27).

*Curiously, the data presented in this manuscript appears to be in contradiction to the scRNA-seq data published by the main principal authors a few years ago (Zappia et al doi 10.15252/embr.201949555 ):
1. In that previous published study, the authors found that Argk1 was a marker for DFM myoblasts (Figure 3B) and the Argk1-GFP labelling restricted to Cut (DFM) myoblasts (Figure 3G). In this study, ArgK1 is now in most all myoblasts (Figure 1A,B)*

**Response:** The reviewer asks for clarification about the expression of Argk1 in DFM and IFM as observed by immunofluorescence and in scRNA-seq data. We carefully analyzed the Argk1 expression using the Argk1::GFP reporter throughout the myoblast layer. As shown in new panels of Figure 1, Argk1::GFP is found labeling some IFM myoblasts (Figure 1D) and is highly expressed in DFM myoblasts (Figure 1E, Figure 2A). To account for myoblasts being not in a monolayer unlike wing epithelial cells, we have included images spanning z-stack in supplementing file (Video S1). A signal intensity scale for both Cut and Argk::GFP and an outline for DFM were added to Figure 1E. These results are in agreement with the scRNA-seq data that show high level of Argk1 in DFM and lower level in several IFM clusters (Dot plots and violin plots in Figure 5 G,J). This also agrees with the scRNA-seq data reported in our earlier publication (Fig 3B in Zappia et al doi 10.15252/embr.201949555). Thus, Argk1 is highly expressed in DFM (that’s why it was called a DFM marker) and is expressed in a subset of IFM albeit at a lower level. The text was clarified on pages 9,10, and 18.

*2. The published study also performed scRNA-seq (same technique as this study) and found 5 IFM clusters and 2 DFM clusters, whereas now there are 8 IFM clusters and 3 DFM clusters, with no obvious explanation as to why the difference. Furthermore, the published dataset found Him as a DFM and IFM cluster marker, whereas here Him is distinctly an IFM cluster marker (IFM a/b/c). The same for E(spl)M6-BFM amongst others (comparing Fig. 1G and 3B of their published manuscript with Fig. 6B of the submitted manuscript, there are numerous inconsistencies of percent expressed/average expression for the markers shown). How do the authors account for such discrepancies? A large section of Figure 6 is spent in (re)classifying IFM/DFM clusters which the authors already performed previously, using similar tools and lineage tracing experiments. It is unclear what, if anything, this adds to what was already known from their previous study.*

**Response:** We apologize for the confusion with single cell data presentation. We note that our published atlas was built using a Drop-seq technology, while in this work we used a more powerful 10X Genomics platform that captures more genes. To match both datasets, we reanalyzed scRNA-seq performed in this work and annotated all clusters following the previously published reference atlas (Figure 5). In this Figure, we present new dotplots and violin plots showing the expression of biomarkers across the clusters. In general, most biomarkers are consistent across the two datasets. However, there were some differences including novel marker genes for some clusters and the presence of a novel cluster IFM_0 that was missing in the published atlas. This is likely due to technical differences between the two technologies. The 10X Genomics platform is known to have an increased sensitivity in gene capture; thus it was not surprising to us to find rare cell populations and discover new biomarkers in the new dataset. Nevertheless, we found that both datasets match well on a broad level. This is in agreement with other studies that directly compared different platforms (for example: 10.1016/j.molcel.2018.10.020). The section of the Results was adjusted accordingly (pages 17-18).

The reviewer asked about the differences in the expression of biomarkers between the two datasets. New dot plots were generated following the reference atlas annotation. The expression of the biomarkers mentioned by the reviewer, E(spl)m6-BFM and Him, are now more consistent with previously published dot plots. Note that the color intensity in the dot plot reflects z-scored average expression calculated across all clusters included in the plot. The color scale changes if clusters are added or removed from the plot because the scaling is relative to the included clusters.

We agree with the reviewer that cluster classification and lineage tracing experiments are not the main focus of the manuscript and therefore these data were removed from Figure 5.

*As a general comment, the Frolov lab (including Dr Maria Zappia) have produced stunning confocal and electron microscopy images in the past looking at muscle development. In comparison, I find the images to be of much poorer quality in this presented study – perhaps simply PDF compression issues? Transmission electron microscopy images would perhaps also be beneficial at myoblast and certain pupal stages of muscle development?*

**Response:** Higher resolution images are now included in Figure 1 and Figure 6.

**Reviewer #2:** *This manuscript is addressing the function of Arginine kinase 1 (Argk1) during Drosophila adult muscle development. The authors find that Argk1 is highly expressed in developing muscles and knock-down of Argk1 in myoblasts results in severe muscle development defects, with defects in myofibril assembly, developmental contractions and mitochondria shape causing pupal lethality. This is explained by reduced ATP levels and changed NAD/NADH ratio. Hence muscles likely have an energy deficit, remain small, and show multiple severe morphology defects.
Single cell analysis shows that Argk1 is not important for myoblast cell fate, which is not surprising given the molecular identify of Argk1, but rather impacts some energy metabolism pathways in the myoblasts, which likely causes severe mitochondria shapes defects and the energy defects.
Overall, I think this is an interesting paper, because the metabolism of cell types, including developing muscles, has not been explored extensively in Drosophila. Although the result that knock-down of Argk1 leads to lethality and metabolic defects is not too surprising, given the likely role of the protein. That said the authors mainly document their findings well. Genetics is well controlled and most phenotypes are properly quantified, statistical tests were applied appropriately. However, in my opinion when studying Argk1, the mitochondria should be better analysed and not only appear in the last figure.
Hence, I support publication of this paper and give the following suggestions to improve the presentation and conclusions of the paper.*

**Response:** We appreciate the reviewer’s comments and thank for the feedback and suggestion to analyze the mitochondria in more details in Argk1-depleted muscle.

*Major points.
1. In mammals Creatine kinase is one main energy buffer, and as far as I know mitochondrial CK is involved in producing phospho-creatine in the intermembrane space, using the ATP being transported out of the matrix by the ATP/ADP carrier. ATP then can diffuse/be transported through the outer mitochondria membrane and is converted to ATP by a different cytoplasmic creatine kinase. But insects do not have creatine kinases. Hence, it might be informative to discuss if Argk1 is the replacement of one or both. Hence, it might be interesting to discuss this in more detail in the intro, possibly adding a scheme, which already highlights a possible role of mitochondria, at the moment they are not mentioned in the introduction at all.*

**Response:** As the reviewer suggested, we included background on creatine kinase function in the Introduction (pages 6-7) and further expanded the discussion of creatine kinases and their isoforms on page 25. Also, we examined the expression of all four genes contained in the arginine kinase gene group and found that *Argk1* is the sole gene expressed in the flight muscle during development (Figure 1B). Additionally, the different isoforms for Argk1 were reported in Figure 1C and their localization was discussed on pages 25-26. Finally, we suggested a possible model for Argk1 function in the developing flight muscles that is aligned with previously published data and the function of creatine kinase at the end of the Discussion on page 27.

*2. The pattern of Argk1 protein localisation in IFMs at 30h and 90h APF strongly reminds me of mitochondria. However, the authors do not seem to consider this possibility. Is Argk1 is enriched in mitochondria, like the mt-CK in mammals (see point 1)? To test this, the authors could express a mitochondria matrix or outer membrane marker (Tom20), for both are UAS-driven red fusion proteins available, combined with Argk1-GFP and do high-resolution confocal microscopy. At the minimum a co-stain with anti-ATPase should be tried, but this might only work well at a late and not early developmental stage. This will address where Argk1 is actually located in the developing muscles and likely highlight the role of Argk1 in mitochondria.*

**Response:** We appreciate the reviewers’ suggestion. We performed the experiments to examine the subcellular localization of Argk1 using the Argk1::GFP reporter and the mitochondrial marker mCherry.mito.OMM. As shown in a new Figure 1, Argk1 indeed localizes to the mitochondria at 24, 45, 72, 90 h APF in the developing IFM and at 1- and 11 day-old adults in mature IFM, along with developing DFM at 90h APF (Figure 1G,J,L-M). These results are described on page 9.

*3. The presented analysis of mitochondria morphology is was unfortunately quite superficial and comes very late in the paper, done only with the anti-ATPase antibody and concludes only with a qualitative statement “elongated vs segmented”, not sure what the latter means. This can be done much better by segmenting mitochondria shapes at late stage pupae using fluorescent markers like Tom20-fluorecent fusions. Likely, even more interesting, would be to look earlier in developing flight muscles, as the likely secondary defects of sarcomere assembly will impact mitochondria morphology in mature muscles. So, investigating mitochondria morphology upon Argk1 knock-down in myoblasts or 30 h APF myotubes, at high resolution, will provide insight closer to the origin of the phenotype.*

**Response:** We thank the reviewer for this suggestion. Data including a detailed examination of sarcomere assembly and maturation along with mitochondrial morphogenesis in developing IFM staged at 27, 46, 72 and 90 h APF are included in Figure 6. Also, data regarding defect in mitochondria structure was moved to Figure 3 and described earlier in the manuscript. Results and conclusions were adjusted accordingly (pages 13, 19-20, 25-27).

*4. It is OK to measure cell size of dissociated myoblasts from wing discs with FACS scattering. However, it would be more elegant to this genetically in vivo, comparing wild type to knockdown. This should be doable when expressing a cell surface marker like CD8-GFP, maybe in a clonal way using a flip-out construct that will only label single cells with GFP while knock-down can still be driven with GAL4 in all cells. The latter needs many crosses and might be beyond what the authors want to invest, however, I would find it neat.*

**Response:** The reviewer suggests quantifying the change in cell size by examining the clones of Argk1-depleted myoblasts in the wing discs. Although such approach works well in epithelial tissues, like wing or eye imaginal discs, where wild type and mutant cells are positioned next to each other in the same plane, it will not be reliable in myoblasts because they do not form a monolayer. Furthermore, there is significant variability in the cell size of myoblasts as documented in Gunage et al (doi.org/10.7554/eLife.03126). Therefore, we used FACS to quantitatively measure cell size. This approach is widely used in the cell cycle field to provide robust data on the role of E2F, RBF, cycD/cdk4 and others in cell growth (for example: Neufeld et al, 1998, Cell, DOI: 10.1016/s0092-8674(00)81462-2; Datar et al, 2000, EMBO J. DOI: 10.1093/emboj/19.17.4543). Notably, using FACS allowed us to compare the size of cells in the same stage of the cell cycle. This is important because myoblasts grow as they undergo differentiation and progress through cell cycle phase. To illustrate the challenges of comparing the cell size by immunofluorescence new images of myoblasts labeled with CD8-GFP are included in Figure 4I. Also, we included a new plot to improve data visualization (Figure 4M) and text was clarified on page 15-16.

*5. Are mitochondria-related GO-terms changed when comparing the mRNA expression between wild-type and knock-down? Ideally, the authors would measure ATP flux, but this is not easy.*

**Response:** No mitochondria-related GO terms were enriched between Argk1-depleted and control myoblasts. In fact, very few genes were significantly changed when an adj-p value was used. As described in response to Reviewer 1 question, we removed GOBP analysis from the manuscript and present volcano plots with differentially expressed genes in Figure S4. Our results and conclusions were adjusted accordingly on page 19.

*Minor.
1. Red-green fluorescent images could be changed to magenta-green.*

**Response:** Images in Figure 1, 3, 4, 7, and S2 have been changed. New images in Figure 1 and 6 are all in magenta-green.

*2. I am unable to see how the genotypes in the UMAP plot of Figure 6A are colour coded. I can see the identity of the different clusters but cannot see which cells are wild-type or knock-down. I can only see this in 6L, but the legend says it should be there in 6A, too.*

**Response:** A new UMAP was generated and split per genotype (Figure 5E). Additionally, a split across replicates is also included in Figure S3B.

*3. LamininC is shown prominently in Figure 6F but not mentioned in the text anywhere unless I missed it.*

**Response:** LamC is a biomarker for IFM_3 and is now included in the text (page 17). We apologize for the omission.

**Reviewer #3:** *General: A prior scRNAseq study by these authors (Zappia et al 2020) of gene expression in Drosophila adult muscle precursor cells (AMPs) revealed ArgK expression, and also that ArgK knockdown through a muscle driver (Mef2Gal4) and UAS ArgK RNAi resulted in lethality. From other work, ArgK is known to function in “maintaining energy homeostasis during different types of stress”.
Building on these findings, the submitted study explores the phenotype resulting from muscle knockdown of ArgK. For a genetic study it is important to justify the tools used: they knockdown ArgK gene function with two pre-existing RNAi lines (with four pre-existing Gal4 driver lines). They then use a broad range of assays to assess muscle development, and differentiated muscle structure and function. They uncover a novel, as far as I am aware, effect of Argk knockdown on energy homeostasis as the flight muscles near completion of their development, and on flight muscle structure and function.

Two General comments:
(1) – the authors should be more precise in the conclusions drawn from some of their experimental results, i.e. don’t over-interpret. See examples below.
(2) - The authors refer several times to “differentiating” and “differentiated” myoblasts (Abstract, Introduction, scRNAseq section in Results). It would be helpful to explain how they categorise as “differentiating” and “differentiated”.*

**Response:** We thank the reviewer for providing constructive feedback. We revised the manuscript to be more precise in conclusions and be more consistent with the terminology.

*Results
Fig 1: They use a GFP protein trap line to assess ArgK expression. They find “strong GFP expression in most of Ct and Twi positive myoblasts”. It would be helpful to explain the difference with a previous paper of theirs (Zappia et al 2020), in which they refer to ArgK as a “DFM marker” in the myoblast population.
Do they have corroborative evidence for “we concluded that Argk1::GFP accurately reflects the endogenous expression of Argk1”, in addition to the observed loss of GFP signal in myoblasts following Argk knockdown?*

**Response:** The reviewer asks for the explanation about the expression of Argk1 in DFM and IFM as detected by immunofluorescence and in scRNA-seq data. We carefully analyzed the Argk1 expression using the Argk1::GFP reporter throughout the myoblast layer. As shown in new panels of Figure 1, Argk1::GFP is found labeling some IFM myoblasts (Figure 1D) and is highly expressed in DFM myoblasts (Figure 1E, Video S1 and Figure 2A). A signal intensity scale for both Cut and Argk1::GFP and an outline for DFM were added to Figure 1E. These results are in agreement with the scRNA-seq data that show high level of Argk1 in DFM and lower level in several IFM clusters (Dot plots and violin plots in Figure 5 G,J) as well as with our earlier publication (Fig 3B in Zappia et al doi 10.15252/embr.201949555). Thus, Argk1 is highly expressed in DFM (that’s why it was called a DFM marker) and is expressed in a subset of IFM albeit at a lower level. Text was clarified on pages 9 and 18.

According to the genomic position of the transgene, Argk1::GFP is not reporting the expression of all Argk1 isoforms as detailed in the new panel in Figure 1C. Because the GFP signal is lost in Argk1 RNAi, we conclude that Argk1::GFP reflects the endogenous expression of, at least, two Argk1 isoforms, but likely not all isoforms. Text was revised on page 9 and 11 to account for this.

*Fig 1C result: “Argk1::GFP is strongly expressed in both fusing myoblasts and multinucleated myotubes at 20 h APF, near the immature myofibrils in the myofibers at 30 h APF, and in between myofibrils in the mature muscles at 96 h APF”. Labelling these cells on the Figure, and having a single channel image for phalloidin, would help ensure that the reader can see what the authors are describing. As presented, it is not clear.*

**Response:** New stainings that include markers like twi-LacZ, Mef2>mCherry.mito.OMM, Futsch, Mhc and Kettin and the single channel images are included in new panels for Figure 1. Text was revised on pages 9-10.

*As a way of justifying their use of the two Argk RNAi lines, could the authors clarify previous characterisation of the RNAi lines they used?
The RNAi line that gives the stronger phenotype does not appear to have been used in either of the two papers Zappia et al cite as previously using ArgK lines. Zappia et al refer to the line as UAS-Argk1-RNAiJF02699 P{TRiP.JF02699}attP2. When I looked it up at BDSC, it said it was stock number 31959. Whereas the two stocks in ref 17 (Justo et el) are stock numbers 41697 and 35221.
The RNAi line that gives the weaker phenotype has been used in Bozzato et al (ref 19). However, it is not clear which line from Bozzato et al, because this paper used two VDRC lines, 34036 and 34037. I realised that the VDRC stock Zappia et al call GD10436, is in fact one of those in Bozzato et al. However, somewhat confusingly the “10436” in the paper under review is the construct ID rather than the fly line ID (either 34036 or 34037).*

**Response:** We apologize for the confusion. All stock numbers were added to the Methods section on page 30. Text was clarified accordingly on page 11. Because both stocks from VDRC (34036 and 34037) were generated with same construct (GD10436), we do not consider these lines as independent stocks. Only experiment in Figure 2B used both VDRC stocks and it is labeled with construct ID and stock number. All other experiments used GD10436/34036. This information is now included in Methods section (page 30).

*Fig 2: The authors “concluded that ArgK is essential throughout muscle development”.
Can the conclusion be more precise? They haven’t addressed in any detail when during development. Do they mean development of just the adult musculature, or do they mean the larval musculature also?
The main driver they use in the paper is Mef2-Gal4, which drives expression throughout embryo development (except the first few hours). Does the difference in phenotype between the Mef2Gal4 and the 1151Gal4 suggest ArgK functions in making the larval musculature, including during embryogenesis?*

**Response:** Given the dynamic expression of *Argk1* mRNA during IFM development (Figure 1B) and the defect in myofibrillogenesis shown when Argk1 is depleted with Act88F-GAL4, a specific driver for IFM at the onset of myofibrillogenesis (Figure 7), our data support a role for Argk1 during myofibrillogenesis in IFM development. Thus, we have re-arranged the manuscript by moving some of the data to Figure 7 and adding new data to address Argk1 requirement in muscle in a more precise manner. Results on page 12, and pages 21-22, the conclusions in abstract on page 2 and in discussion on page 24 have been revised accordingly.

The differences in the phenotypes observed between Mef2-Gal4 and 1151-Gal4 drivers could be due to 1) the Gal4 expression (how strong Gal4 expression/activity is), 2) the extend of the knock down (meaning muscle type: Mef2-Gal4 is expressed in cardiac, visceral, skeletal muscles, whereas 1151-Gal4 is expressed only in adult skeletal muscles), and 3) developmental timing (embryonic, larval and pupal development). Further examination of the role of Argk1 in larval muscles is outside the scope of this study, which focuses on the flight muscle. Overall, our data support a role for Argk1 in flight muscle development and our conclusions were adjusted accordingly.

*Muscle phenotype structure/function: Up to this point the authors have looked looking at lethality or flight function. Now they look at aspects of muscle development. Parts of this section (Figs 3 and 4) feel somewhat incomplete.

Fig 3 – They start with Mef2 Gal4 driving UAS Argk RNAi and assess the structure (including sarcomere organisation) of the flight muscles (both the IFMs and the DFMs). They quantify a decrease in cross-sectional area and myofibril width with Argk RNAi. However, there is quantification only for the IFMs.

Fig 3D – it would be helpful to have single channel phalloidin for clarity of the results.
They conclude “our findings further support the requirement of Argk1 during the development of both flight muscles”. BUT because the Argk knockdown is from so early in the developmental programme, this result tells you little about which step in the process is actually affected.*

**Response:** The figures were rearranged to account for this. The analysis on the overall mitochondria structure and the data related to ATP content and NAD/NADH ratio are included in Figure 3 to make it more complete. Single channel phalloidin images are included in Figure 3 too. Text in Results (pages 12-14) and conclusions were revised and adjusted accordingly.

*Fig 4 – They then use Act88F Gal4 to assay later in development (it is expressed from 20h after pupal formation), but with the “strong” UAS Argk RNAi line only.
They do the same muscle structure assays as with Mef2Gal4, but this time only assess the IFMs (and not the DFMs). They observe a decrease in IFM cross-section area with Argk RNAi. This appears to be less than with Mef2Gal4, but this is not commented upon.
Sarcomere length is measured here, although it doesn’t appear to have done for Mef2Gal4 in Fig 3 (although sarcomere length is measured in Fig S1 for Mef2Gal4 driving the “weaker” Argk RNAi line).
Strangely in Fig 4 is it DLM-IFM muscle 4 that is quantitated, whereas it was muscle 3 in Fig 3.
This may matter as there is evidence that the different muscles of the DLM-IFMs may not respond in the same way, e.g. see Chaturvedi et al 2019 (http://dx.doi.org/10.1098/rsob.190087)*

**Response**: The expression of Act88F-GAL4 is specific to IFM, therefore, we do not expect a phenotype in DFM. That is the reason why the analysis on the DFM was not included when analyzing phenotype with Act88F-GAL4. Text was clarified on page 21.

Sarcomere length could not be assessed in Figure 3 because the defect was too severe. The automated plug-in in ImageJ did not provide reliable and accurate data. That is the reason why we decided not quantify it.

Quantifications for both muscle 3 and muscle 4 is now included in Figure 3, S2 and 7. Text was revised accordingly (pages 12, 22).

*Fig 6 scRNAseq: the Aim was to assess transcriptome effects of ArgK depletion on the wing disc associated myoblasts (they used Mef2 Gal4 and the “stronger” UAS Argk RNAi).
Their analysis divided this cell population into 8 IFM and 3 DFM groups. This is different (has more groups) to their earlier paper (Zappia et al 2020), and it would be valuable to explain the difference.

Then in a bit of a detour from the main topic for the paper, they describe various genes expressed in these defined groups, and follow one, Sp1, for the DFMs by “GTRACE”, identifying likely progenitor cells.

They then compared the AMP transcriptomes in control and Argk RNAi, and concluded that ArgK RNAi doesn’t change AMP “identity” (comparing the myoblast groups and a range of marker genes).*

**Response:** We apologize for the confusion with single cell data presentation. We note that our published atlas was built using a Drop-seq technology, while in this work, we used a more powerful 10X Genomics platform that captures more genes. Even though these two platforms are droplet-based technologies, the 10xGenomics is known to have higher sensitivity in transcript capture and efficiency in cell recovery than Drop-seq. To match both datasets, we reanalyzed scRNA-seq performed in this work and annotated all clusters following the previously published reference atlas (Figure 5E). In this Figure, we present new dot plots and violin plots showing the expression of biomarkers across the clusters. In general, most biomarkers are consistent across the two datasets. However, there were some differences including novel marker genes for some clusters and the presence of a novel cluster IFM_0 that was missing in the published atlas. This is likely due to technical differences between the two technologies, including higher sensitivity of the 10X Genomics platform, as noted above. Nevertheless, we found that both datasets match well on a broad level. This is in agreement with other studies that directly compared different platforms (for example: 10.1016/j.molcel.2018.10.020). The section of the Results was revised accordingly (page 18).

We agree with the reviewer. It is kind of a detour and that’s the reason why we decided to withdraw the additional data, including marker validation. It distracts from the main point of the manuscript. We are currently preparing a manuscript that will include a higher resolution scRNA-seq integrated with ATAC-seq.

*Fig 7 - However, a GO analysis revealed range of energy/metabolic changes in Argk knockdown. They therefore assessed ATP level, NAD+/NADH ratio and mitochondrial morphology. They did this only at the pharate stage, and with just one Gal4 driver (Mef2Gal4) and just the stronger UAS ArgK RNAi line. With Argk knockdown they found: low ATP level, altered NAD+/NADH ratio and abnormal morphology of the mitochondrial network. (n.b. Fig S3 has mitochondrial morphology results for the “weaker” UAS Argk RNAi line).

A conclusion in the Introduction is: “our work reveals a novel function of Argk1 during muscle development through its regulation of energy metabolism.” However, this seems an overstatement. Firstly, aspects of energy homeostasis are only assessed at the pharate stage, whereas the strongest developmental effects were from Argk RNAi expression driven from the earliest stages of adult muscle progenitor cells (Mef2Gal4), or others from quite young pupae (Act88F). Secondly, a causal link is not established. As it stands, it is a correlation, the authors have not clearly linked energy and muscle development. Moving beyond this would be a valuable addition.*

**Response:** We agree with the reviewer. The energy homeostasis data was moved to Figure 3 where the phenotype at pharate stage with Mef2-Gal4 were assessed. Also, text in the manuscript was revised to adjust the conclusions in the title, abstract, introduction (pages 7-8), results (page 14) and discussion (pages 23, 25, 27).

*The authors make similar comments to this in both the Abstract and the Discussion, both times saying Argk function “throughout muscle development”. Is the Argk function required throughout development, or are just at some times that have consequences later? The temporal aspect is addressed to some extent using different Gal4 drivers to induce ArgK RNAi expression, but these are not sufficient to say throughout development?*

**Response:** We present new data showing the staining of the Argk1-depleted muscles at 27, 46, 72 and 90 h APF with mito-GFP, Mhc and Kettin in Figure 6. This allowed us to carefully examine the defects in myofibrillogenesis during IFM development. Our data support the idea that Argk1 plays an important role in myofibrillogenesis in during IFM development. The text was revised accordingly in the title, abstract, results (page 20), discussion (pages 23, 24).

*Spontaneous contractions Fig 7: “We did not detect a single twitching event in Mef2>Argk1- RNAi animals (Figure 7H, Video S2). Significantly, the animals were alive at that time point since very mild movements across all flight muscles were readily observed occasionally.” Can the authors say a bit more on this? It is obviously an important point, as they know the lethality period is sometime during pupation.*

**Response:** Animals do not die until they reach the pharate stage at the end of pupation – around 90-96h APF. Recording of muscle twitching were done at 48h APF in alive animals. We confirmed animals were alive because mild movements were detectable. However, flight muscles were not contracting. Text was revised on page 21.

*In the Discussion they comment: “we found that Argk1-depleted muscles completely lack such twitching events and, concordantly, have abnormal sarcomere structures. This finding suggests that Argk1 is required for spontaneous muscle twitching during development presumably by supporting high energy demand during contractions”. The authors could consider whether this is not the explanation and, perhaps this functional deficit might be the consequence of a prior development defect.*

**Response:** We appreciate the comment. Our interpretation was based on recent studies from Spletter and Schnorrer laboratories ([doi.org/10.1016/j.cub.2024.07.079](https://doi.org/10.1016/j.cub.2024.07.079), [doi.org/10.7554/eLife.34058](https://doi.org/10.7554/eLife.34058), [doi.org/10.1242/dev.140723](https://doi.org/10.1242/dev.140723)) demonstrating the importance of muscle twitching in myofibrillogenesis and mitochondrial morphology. In fact, one of the strongest phenotypes in Argk1 depleted muscle is the lack of muscle twitching. However, we agree with the reviewer’s point. Text was revised on page 26 to acknowledge this.

*Minor:
Introduction paragraph 2 “muscle-specific knockdown of one of these genes, Arginine kinase 1 (Argk1), resulted in pupal lethality, ” – this needs a supporting reference.*

**Response:** Reference was added in page 6.

*Results: “Quantifications confirmed that the cross-sectional area of muscle 3 and 4 from DLM-IFM were statistically significantly smaller than in wild type control (Figure 3C).” Fig 3C only has muscle 3.*

**Response:** Quantification for both muscle 3 and 4 are now included.

*Results: “thus indicating that the line JF02699 had a stronger phenotype than GD10436.” – this was said already in Fig1-linked text.*

**Response:** Text was revised on page 13.

*Results cell size: “The wing discs from Mef2>mCherry-RNAi htl>CD8-GFP and Mef2>Argk1-RNAi htl>CD8-GFP animals were dissociated into single cell suspension” genotype is missing a “;”.*

**Response:** We apologize for the omissions. Text was revised accordingly on page 16.
