# Supplementary material for: Arginine Kinase 1 supports energy homeostasis in Drosophila flight muscle development
Source: PLoS Genet. 2026 Sep 17;22(9):e1012304. doi: 10.1371/journal.pgen.1012304 (PMC13585230; doi:10.1371/journal.pgen.1012304)
Supplement: Supplementary file 2 [file pgen.1012304.s018.docx]

PGENETICS-D-26-00192R1

**Reviewers' comments:**

Reviewer's Responses to Questions

**Comments to the Authors:
Please note here if the review is uploaded as an attachment.**

***Reviewer #1:*** *The study of the role of Argk1 in flight muscle development by Zappia and colleagues has been substantially revised, not only with additional data, but also in a substantial rewrite to present a more logical study of the role of this important enzyme during myogenesis. They have addressed most of my concerns, especially in the explanation of what appeared to be inconsistencies in the scRNA-seq data and have made it much clearer in both the results and discussion of the different isoforms that exist for Argk1. That said, given that Z-band formation is severly affected by Mef2>Argk1 RNAi during early stages of sarcomerogenesis (Fig 3D, anti-Kettin), but unaffected by RNAi at later stages with Act88F>Argk1 RNAi (Fig 7E, anti-Kettin), this suggests that the Z-band-located shorter isoform plays an energentic role in early stages of sarcomerogenesis. While this is briefly discussed, citing the previous data, no experiments are performed eg antibody localization of the protein to the z-band, RNAi against only the longer, mitochondrial isoforms. While the manuscript is now more focussed on the mitochndrial role, this perhaps merits greater reflection and discussion. Is the mitochondrial role in energy homeostasis restricted to the flight muscle stages or is it more widespread? Knocking down Argk1 only at pharate stages and continued into adulthood through Gal80 control (eg Mef2Gal4,Gal80ts) should lead to flightless adults if this is the case.
Overall, the new data and substantial rewriting of the manuscript provide a much clearer picture of the importance of Argk1 in muscle development and the authors are more cautious in not overinterpreting their data.*

**Response:** We thank the reviewer for the positive feedback and the suggestions. We agree that further investigation of the differential roles of the Argk isoforms is warranted. However, we think it will be out of the scope of this manuscript. The proper analysis will require both the optimization of the thermosensitive system and the generation of new tools, which includes isoform-specific knockdowns and reporters. We understand the relevance of this point, but, it is not something that can be readily added into the revision.

***Reviewer #2:*** *I am happy to see that this revised manuscript by Zappia and colleagues now convincingly documents the localisation of Argk1 to mitochondria in myoblasts, developing myotubes and also mature flight muscle fibres. As this is now shown in Figure 1 (and not Figure 7), one can now understand the main message of the paper. The new images are beautiful!
Also, cell size was tried to assess in vivo and is shown. I agree that measuring size by FACS makes sense.
Further, biochemical background knowledge of Argk1 function was added.
Finally, mitochondria morphology is now shown throughout development with appropriate marker. The quantification, elongated vs segmented, is still not very quantitative, but the phenotype is clear, hence it would likely not add significantly to the paper to segment individual mitochondria shapes, which might be tricky in the KD.
In total, most of my suggestions have been addressed, hence I recommend publication of this manuscript.*

We thank the reviewer for the positive comments.

*Minor.
1. Typo in Figure 3D, one of the “phalloidin” is missing the “n”.
2. Ref 34 looked at spontaneous twitches in developing abdominal muscles and showed it is important for cross striations, which these muscles, but not the IFMs, form. To my knowledge it was not carefully investigated if the spontaneous twitches in developing IFMs until 48h APF play an instructive role in morphogenesis. There might be data on motor neuron KO but not sure if this stops the twitching. Hence, authors might want to tone down the relevance statement. Or mention the other muscles.*

**Response:** Typo was corrected and statements were revised in the abstract (page 2), author summary (page 3), introduction (page 6), results (page 19-20), and discussion (page 25).

***Reviewer #3:*** *Due to time-pressure I am only commenting to the authors’ responses to the points I raised in my initial review.
I will comment on each of the authors’ responses in turn.

Fig1
The text on the revised p9 doesn’t have a reference for the scRNAseq results, it should have.*

**Response:** The reference for scRNA-seq [10] is included at the end of the sentence on page 8.

*It is a moot point whether higher expression of ArgK in DFMs than in IFM progenitor cells makes it a “DFM marker”.
Argk1::GFP: The authors indicate that the text was revised on pages 9-10 to account for this. The meaning of the new sentence on page 11 (“Yet, the GFP signal was mainly detected in mitochondria, likely reflecting that the GFP trap line reflects the expression of the sole isoforms Argk1-A and Argk1-E”) is not clear.*

**Response:** Text on page 9 was revised to make it clearer.

*Re. my Fig 1C comment on Argk1::GFP expression: I couldn’t see in the modified Fig 1 either the asked-for labelling of expression, or the single channel for phalloidin.*

**Response:** Figure 1 in our revised submission was extensively modified because we perfomed a high number of new immunofluorescence and numerous new images were added, thus the original panel Fig 1C was split across Figure 1 and Figure S1B. We included phalloidin to visualize the overall morphology of myotubes. The single-channel phalloidin is shown with the non-overlapping dye DAPI, and therefore, myotube structure is well visible. For this reason and due to space-constrains in Figure 1, the single-channel phalloidin is presented in Figure S1B and labeling is added in Figure 1H,I,J to mark myotubes as requested.

*The authors have clarified the two VDRC ArgK RNAi stocks used as requested.

I previously commented: “The RNAi line that gives the stronger phenotype does not appear to have been used in either of the two papers Zappia et al cite as previously using ArgK lines…...”
Although the authors do not directly address my comment, they do now have a reference in the Methods to the stronger RNAi line, and no longer cite the two papers in the original submission they cited as previously using ArgK lines.

The authors have responded to my comment linked to Fig 2.
They have modified their conclusion in the original m/s that “ArgK is essential throughout muscle development” in the Abstract, p12 and Discussion. However, this does make the conclusion from this section of their work less striking.

Fig 3: the authors have added single channel phalloidin as asked for.

Fig 3: The text on new p14 says “Notably, the Argk1-depleted DFM and IFM were significantly reduced in size.”
However, there is still only quantification for (part of) the IFM, so there is no quantification for the DFMs, therefore one cannot say that the Argk1-depleted DFMs are “significantly reduced in size”. This should be modified.*

**Response:** Text on page 11 was revised accordingly. The word “significantly” was removed.

*Fig4: the authors have now included quantification of both muscle 3 and 4.

Fig 6 scRNAseq - the relevant section of the Results has been modified.
However, the response from the authors really doesn’t make it clear why the IFM and DFM myoblast group classification is different in their initial submitted version to PLoS Genetics when compared to their previously published classification.
It also isn’t made clear why they have now dropped their initially submitted IFM and DFM classification.
It also isn’t clear why the classification in this modified version (5 IFM groups and 1 DFM) is different to both the initially submitted version and the previously published classification.
They seem to say that the 10x Genomics sequencing methodology in this paper is more powerful than their previously used “DropSeq”.
As 10x Genomics is “more powerful”, it is not apparent why they now drop the classification of 8 IFM groups plus 3 DFM groups derived from the 10x Genomics results.
Taken together it makes one question the significance of these groups as presented.*

**Response:** We understand the confusion and apologize for not making it clearer. The reason why the scRNA-seq has undergone significant modification is based on the requests from the reviewer #1 and the editors. They suggested to reconcile our data with the previously published Drop-seq dataset. That is why we ended-up merging back the clusters to match it with the published atlas. Some of the new clusters in the original version are subclusters of the published Drop-seq atlas. For example, the clusters IFM_b and IFM_c in the originally submitted Figure 6A were merged into IFM_1 cluster as in the Drop-seq atlas. However, we identified a new cluster, IFM_0, that was missed in the Drop-seq dataset.

In the previous “Response to reviewers” we agreed that it was a detour from the main point of the paper and that was the reason why we decided to remove the higher resolution cluster assignment. We are currently working on building a higher resolution scRNA-seq by integrating it with ATAC-seq to confirm the significance of these new clusters.

*Fig 7 comment. The authors have modified the Title, Abstract (however, it does make the conclusion from this section of their work less striking)
The narrative works better now with the energy homeostasis data moved to Figure 3 .
They have also modified the Discussion – however, on the new p30 I don’t think the new sentence at the end of the first paragraph “……. and consequently affects energy homeostasis” is really clear.
Could it not be that ArgK affects energy homeostasis and consequently aspects of the flight muscles?*

**Response:** Text on page 22 was revised accordingly.

*The new results added to Fig 6 are helpful in aspects of ArgK effects on IFM development.

The added muscle-twitching comment is also helpful.

The minor comments are dealt with.*
