# Supplementary material for: Arginine Kinase 1 supports energy homeostasis in Drosophila flight muscle development
Source: PLoS Genet. 2026 Sep 17;22(9):e1012304. doi: 10.1371/journal.pgen.1012304 (PMC13585230; doi:10.1371/journal.pgen.1012304)
Supplement: Peer review history

# Peer review: Decision Letter 0

**DOI:** 10.1371/journal.pgen.1012304.r001

**Authors:** Giovanni Bosco, Lolitika Mandal

16 Apr 2026

PGENETICS-D-26-00192

Arginine Kinase 1 regulates energy homeostasis in Drosophila muscle development

PLOS Genetics

Dear Dr. Frolov,

Thank you for submitting your manuscript to PLOS Genetics. After careful consideration, we feel that it has merit but does not fully meet PLOS Genetics's publication criteria as it currently stands. Therefore, we invite you to submit a revised version of the manuscript that addresses the points raised during the review process.

We have summarized the essential parts of the Reviewer's comments that need to be addressed in the revised version.

1. ***Mitochondrial Localization of Argk1***  : It is essential to probe whether Argk1 is enriched in mitochondria, like the mt-CK in mammals? Co-labelling with a mitochondrial matrix or outer membrane marker, combined with Argk1-GFP, is needed. This data will make it clearer where Argk1 is in developing muscles and probably show how it works in mitochondria during muscle development.

*2. **Mitochondrial morphology** :*  The current analysis of mitochondrial morphology is inadequate. Authors should examine mitochondrial morphology following Argk1 knockdown in myoblasts or 30-hour APF myotubes at high resolution. Such data will be vital for understanding the origin of the observed phenotypic changes.

*3. **Clonal Analysis***   for Cell Size Variations must be conducted to compare cell size differences, thereby enhancing the robustness of their findings.

*4. **Regulation of Oxidoreductase Enzymes***  : Why Argk1 knockdown would lead to transcriptional up- or downregulation of oxidoreductase enzymes is unclear. Authors are advised to knock down these oxidoreductase enzymes and assess whether such genetic perturbation phenocopies the muscle phenotypes observed with Argk1.

*5. **Distinct Functions of ArgK1***  : Authors need to address whether the muscle and mitochondrial phenotypes reflect two distinct functions for ArgK1 – one in the mitochondria and the other at the Z-line in the developing myofiber.

*6. **Inconsistencies in Data Presentation***  **:**  As the reviewers noted, numerous inconsistencies need to be resolved. For example, the number of clusters of IFM and DM, and the different markers, creates confusion in interpreting single-cell transcriptomic data. It is essential to clarify these discrepancies.

*7. **Temporal Dynamics of Argk1:GFP** :*  The Argk1:GFP is dynamically expressed over time. Co-labelling with markers for myoblasts, myotubes, myofibers, and mature muscle is lacking. Performing these additional co-labelling experiments is essential.

*8. **Clarification of Argk 1's Role***  : The authors have “concluded that ArgK is essential throughout muscle development”. Yet this statement does not fully align with the data presented. Do they mean development of just the adult musculature, or do they mean the larval musculature also?

*9. **Comprehensive Analysis of Muscle Development***  : As noted by the reviewers, there is a lack of comprehensive analysis regarding various aspects of muscle development. Several quantifications are missing, and although multiple Gal4 drivers are used, the results need to be cohesively integrated to enhance understanding.

*10. **Energy metabolism and Muscle development***  **:**  the authors should be more precise in the conclusions drawn from some of the experimental results. For example: “our work reveals a novel function of Argk1 during muscle development through its regulation of energy metabolism.” The aspects of energy homeostasis are assessed only at the pharate stage. In contrast, the strongest developmental effects were from Argk RNAi driven from the earliest stages of adult muscle progenitor cells (Mef2Gal4) or from others in quite young pupae (Act88F). The authors should clearly link energy and muscle development.

Please submit your revised manuscript by Jun 15 2026 11:59PM. If you will need more time than this to complete your revisions, please reply to this message or contact the journal office at plosgenetics@plos.org. When you're ready to submit your revision, log on to https://www.editorialmanager.com/pgenetics/ and select the 'Submissions Needing Revision' folder to locate your manuscript file.

Please include the following items when submitting your revised manuscript:

* A letter that responds to each point raised by the editor and reviewer(s). You should upload this letter as a separate file labeled 'Response to Reviewers'. This file does not need to include responses to any formatting updates and technical items listed in the 'Journal Requirements' section below.

* A marked-up copy of your manuscript that highlights changes made to the original version. You should upload this as a separate file labeled 'Revised Manuscript with Track Changes'.

* An unmarked version of your revised paper without tracked changes. You should upload this as a separate file labeled 'Manuscript'.

If you would like to make changes to your financial disclosure, competing interests statement, or data availability statement, please make these updates within the submission form at the time of resubmission. Guidelines for resubmitting your figure files are available below the reviewer comments at the end of this letter.

We look forward to receiving your revised manuscript.

Kind regards,

Lolitika Mandal, Ph.D

Academic Editor

PLOS Genetics

Giovanni Bosco

Section Editor

PLOS Genetics

Aimée Dudley

Editor-in-Chief

PLOS Genetics

Anne Goriely

Editor-in-Chief

PLOS Genetics

**Journal Requirements:**

If the reviewer comments include a recommendation to cite specific previously published works, please review and evaluate these publications to determine whether they are relevant and should be cited. There is no requirement to cite these works unless the editor has indicated otherwise.

1) Please ensure that the CRediT author contributions listed for every co-author are completed accurately and in full.

At this stage, the following Authors/Authors require contributions: Maria Paula Zappia, Anton Westacott, Hannah Cooke, Rhianna Geary, Libby Travers, Lucia de Castro, Oliver Carty, and Maxim V Frolov. Please ensure that the full contributions of each author are acknowledged in the "Add/Edit/Remove Authors" section of our submission form.

The list of CRediT author contributions may be found here: https://journals.plos.org/plosgenetics/s/authorship#loc-author-contributions

2) Please provide an Author Summary. This should appear in your manuscript between the Abstract (if applicable) and the Introduction, and should be 150-200 words long. The aim should be to make your findings accessible to a wide audience that includes both scientists and non-scientists. Sample summaries can be found on our website under Submission Guidelines:

https://journals.plos.org/plosgenetics/s/submission-guidelines#loc-parts-of-a-submission

3) Please upload all main figures as separate Figure files in .tif or .eps format. For more information about how to convert and format your figure files please see our guidelines:

https://journals.plos.org/plosgenetics/s/figures

4) We notice that your supplementary Figures are included in the manuscript file. Please remove them and upload them with the file type 'Supporting Information'. Please ensure that each Supporting Information file has a legend listed in the manuscript after the references list.

5) Please amend your detailed Financial Disclosure statement. This is published with the article. It must therefore be completed in full sentences and contain the exact wording you wish to be published.

1) State the initials, alongside each funding source, of each author to receive each grant. For example: "This work was supported by the National Institutes of Health (####### to AM; ###### to CJ) and the National Science Foundation (###### to AM)."

2) State what role the funders took in the study. If the funders had no role in your study, please state: "The funders had no role in study design, data collection and analysis, decision to publish, or preparation of the manuscript."

3) If any authors received a salary from any of your funders, please state which authors and which funders..

If you did not receive any funding for this study, please simply state: u201cThe authors received no specific funding for this work.u201d

**Reviewers' comments:**

Reviewer's Responses to Questions

**Comments to the Authors:**

**Please note here if the review is uploaded as an attachment.**

**Reviewer #1:**  Zappia and colleagues, analyze the role the Drosophila arginine kinase 1 (Argk1) plays in flight muscle development. The authors’ logic to study Argk1 in this developmental process is that it’s muscle-specific knockdown results in pupal lethality and thus it may have an important function during muscle development. While not untrue, it is a weak basis for its specific study since 1. numerous RNAi screens over many years, some dating more than 15 years, using the same strategy have revealed very many genes with lethal pupal phenotypes and 2. Drosophila flight muscle is not required for viability.

A more logical and valid reason for its study is the role Argk1 plays in energy homeostasis : Argk1 is one of four Drosophila arginine kinases that catalyze the transfer of phosphate from ATP to arginine, yielding phosphoarginine and ADP. They act as 'phosphagen kinases' in invertebrates, playing a key role as ATP buffering systems in cells that display high and variable rates of ATP turnover. As flight muscle is the most energy demanding tissue in Drosophila, the role Argk1 plays is thus potentially of interest.

While the experiments are by-and-large correct and well-performed, I feel the choice of experiments performed to study energy homeostasis are of little use. For example, a very complicated scRNA-seq was performed (Figure 6), whose conclusion was that Argk1 doesn’t generally affect muscle precursor identity. The authors have previously studied transcription factor function in flight muscle development, where scRNA-seq is an appropriate study. It is of little surprise, to this reviewer, that the identity of muscle precursors don’t change when one knocks down expression of a cytoplasmic arginine kinase. A bulk (or better single-cell) metabolomics study would perhaps have been more appropriate for studying the effects of knockdown a such an enzyme?

Since the scRNA-seq approach did not change the identity of the muscle precursors, they analyzed their single cell data as pseudo-bulk RNA-seq for differential gene expression analysis. Here, a very simple an inexpensive bulk RNA-seq would perhaps have yielded higher quality differential gene expression data (or not) permitting a p-value cut off much more stringent than 0.05 (why was p-adj not used?). Nevertheless, some genes were found as differentially expressed although whether upregulated or downregulated was not specified in the gene ontology enrichment analysis which hinted at defects in energy homeostasis. However, while the link is quite weak, metabolomic tests, limited to simple NAD+/NADH ratio and global ATP level tests demonstrated a reduction in both upon Argk1 knockdown. Again, why Argk1 knockdown would lead to the transcriptional up/down regulation of oxidoreductase enzymes is unclear and not discussed. Do knockdown of these oxidoreductase enzymes phenocopy the muscle phenotypes observed with that of Argk1?

The authors then finally link the phenotypes observed upon Argk1 knockdown to mitochondrial network abnormalities to conclude it as an essential enzyme for energy homeostasis during muscle development. While it has been previously shown by Avellaneda and colleagues (doi: 10.1038/s41467-021-22058-7 ) that muscle and mitochondrial development are intricately intertwined, by Zappia and Frolov that factors controlling both myogenesis and mitochondrial biogenesis can affect both muscle and mitochondria morphologically (doi 10.1038/ncomms10509 ), the study by Poliacikova and colleagues (doi 10.1038/s41467-023-38986-5 ) shows that one can affect/eliminate only mitochondrial energy function, without affecting muscle development. Thus, it is extremely difficult to correlate that disruption to energy homeostasis is the reason for the observed impaired myogenesis. Furtheremore, two different ArgK1 isozymes have been reported in Drosophila (Munneke and Collier doi 10.1007/bf00555494 ): a smaller cytoplasmic form that locates at the Z line of myofibrils of IFM and a bigger mitochondrial form that locates in the mitochondrion (Lang et al doi 10.1007/bf00711796 ). Since the difference is in a longer N-terminal sequence, both forms will be affected by the RNAi. Have the authors considered that the muscle and mitochondrial phenotypes simply reflect two distinct functions for ArgK1 – one in the mitochondria and the other at the Z-line in the developing myofiber?

As energy homeostasis is the major interesting aspect of the study, a more in depth metabolomics study is required and/or RNAi of other enzymes required for energy production to try to understand how energy homeostasis is required for myogenesis. At a bare minimum, quantification of phosphorylarginine (the product of reversible transfer of a phosphate between ATP and arginine that is catalyzed by ArgK1) should be performed.

Curiously, the data presented in this manuscript appears to be in contradiction to the scRNA-seq data published by the main principal authors a few years ago (Zappia et al doi 10.15252/embr.201949555 ):

1. In that previous published study, the authors found that Argk1 was a marker for DFM myoblasts (Figure 3B) and the Argk1-GFP labelling restricted to Cut (DFM) myoblasts (Figure 3G). In this study, ArgK1 is now in most all myoblasts (Figure 1A,B)

2. The published study also performed scRNA-seq (same technique as this study) and found 5 IFM clusters and 2 DFM clusters, whereas now there are 8 IFM clusters and 3 DFM clusters, with no obvious explanation as to why the difference. Furthermore, the published dataset found Him as a DFM and IFM cluster marker, whereas here Him is distinctly an IFM cluster marker (IFM a/b/c). The same for E(spl)M6-BFM amongst others (comparing Fig. 1G and 3B of their published manuscript with Fig. 6B of the submitted manuscript, there are numerous inconsistencies of percent expressed/average expression for the markers shown). How do the authors account for such discrepancies? A large section of Figure 6 is spent in (re)classifying IFM/DFM clusters which the authors already performed previously, using similar tools and lineage tracing experiments. It is unclear what, if anything, this adds to what was already known from their previous study.

As a general comment, the Frolov lab (including Dr Maria Zappia) have produced stunning confocal and electron microscopy images in the past looking at muscle development. In comparison, I find the images to be of much poorer quality in this presented study – perhaps simply PDF compression issues? Transmission electron microscopy images would perhaps also be beneficial at myoblast and certain pupal stages of muscle development?

**Reviewer #2:**  This manuscript is addressing the function of Arginine kinase 1 (Argk1) during Drosophila adult muscle development. The authors find that Argk1 is highly expressed in developing muscles and knock-down of Argk1 in myoblasts results in severe muscle development defects, with defects in myofibril assembly, developmental contractions and mitochondria shape causing pupal lethality. This is explained by reduced ATP levels and changed NAD/NADH ratio. Hence muscles likely have an energy deficit, remain small, and show multiple severe morphology defects.

Single cell analysis shows that Argk1 is not important for myoblast cell fate, which is not surprising given the molecular identify of Argk1, but rather impacts some energy metabolism pathways in the myoblasts, which likely causes severe mitochondria shapes defects and the energy defects.

Overall, I think this is an interesting paper, because the metabolism of cell types, including developing muscles, has not been explored extensively in Drosophila. Although the result that knock-down of Argk1 leads to lethality and metabolic defects is not too surprising, given the likely role of the protein. That said the authors mainly document their findings well. Genetics is well controlled and most phenotypes are properly quantified, statistical tests were applied appropriately. However, in my opinion when studying Argk1, the mitochondria should be better analysed and not only appear in the last figure.

Hence, I support publication of this paper and give the following suggestions to improve the presentation and conclusions of the paper.

Major points.

1. In mammals Creatine kinase is one main energy buffer, and as far as I know mitochondrial CK is involved in producing phospho-creatine in the intermembrane space, using the ATP being transported out of the matrix by the ATP/ADP carrier. ATP then can diffuse/be transported through the outer mitochondria membrane and is converted to ATP by a different cytoplasmic creatine kinase. But insects do not have creatine kinases. Hence, it might be informative to discuss if Argk1 is the replacement of one or both. Hence, it might be interesting to discuss this in more detail in the intro, possibly adding a scheme, which already highlights a possible role of mitochondria, at the moment they are not mentioned in the introduction at all.

2. The pattern of Argk1 protein localisation in IFMs at 30h and 90h APF strongly reminds me of mitochondria. However, the authors do not seem to consider this possibility. Is Argk1 is enriched in mitochondria, like the mt-CK in mammals (see point 1)? To test this, the authors could express a mitochondria matrix or outer membrane marker (Tom20), for both are UAS-driven red fusion proteins available, combined with Argk1-GFP and do high-resolution confocal microscopy. At the minimum a co-stain with anti-ATPase should be tried, but this might only work well at a late and not early developmental stage. This will address where Argk1 is actually located in the developing muscles and likely highlight the role of Argk1 in mitochondria.

3. The presented analysis of mitochondria morphology is was unfortunately quite superficial and comes very late in the paper, done only with the anti-ATPase antibody and concludes only with a qualitative statement “elongated vs segmented”, not sure what the latter means. This can be done much better by segmenting mitochondria shapes at late stage pupae using fluorescent markers like Tom20-fluorecent fusions. Likely, even more interesting, would be to look earlier in developing flight muscles, as the likely secondary defects of sarcomere assembly will impact mitochondria morphology in mature muscles. So, investigating mitochondria morphology upon Argk1 knock-down in myoblasts or 30 h APF myotubes, at high resolution, will provide insight closer to the origin of the phenotype.

4. It is OK to measure cell size of dissociated myoblasts from wing discs with FACS scattering. However, it would be more elegant to this genetically in vivo, comparing wild type to knockdown. This should be doable when expressing a cell surface marker like CD8-GFP, maybe in a clonal way using a flip-out construct that will only label single cells with GFP while knock-down can still be driven with GAL4 in all cells. The latter needs many crosses and might be beyond what the authors want to invest, however, I would find it neat.

5. Are mitochondria-related GO-terms changed when comparing the mRNA expression between wild-type and knock-down? Ideally, the authors would measure ATP flux, but this is not easy.

Minor.

1. Red-green fluorescent images could be changed to magenta-green.

2. I am unable to see how the genotypes in the UMAP plot of Figure 6A are colour coded. I can see the identity of the different clusters but cannot see which cells are wild-type or knock-down. I can only see this in 6L, but the legend says it should be there in 6A, too.

3. LamininC is shown prominently in Figure 6F but not mentioned in the text anywhere unless I missed it.

**Reviewer #3:**  General: A prior scRNAseq study by these authors (Zappia et al 2020) of gene expression in Drosophila adult muscle precursor cells (AMPs) revealed ArgK expression, and also that ArgK knockdown through a muscle driver (Mef2Gal4) and UAS ArgK RNAi resulted in lethality. From other work, ArgK is known to function in “maintaining energy homeostasis during different types of stress”.

Building on these findings, the submitted study explores the phenotype resulting from muscle knockdown of ArgK. For a genetic study it is important to justify the tools used: they knockdown ArgK gene function with two pre-existing RNAi lines (with four pre-existing Gal4 driver lines). They then use a broad range of assays to assess muscle development, and differentiated muscle structure and function. They uncover a novel, as far as I am aware, effect of Argk knockdown on energy homeostasis as the flight muscles near completion of their development, and on flight muscle structure and function.

Two General comments:

(1) – the authors should be more precise in the conclusions drawn from some of their experimental results, i.e. don’t over-interpret. See examples below.

(2) - The authors refer several times to “differentiating” and “differentiated” myoblasts (Abstract, Introduction, scRNAseq section in Results). It would be helpful to explain how they categorise as “differentiating” and “differentiated”.

Results

Fig 1: They use a GFP protein trap line to assess ArgK expression. They find “strong GFP expression in most of Ct and Twi positive myoblasts”. It would be helpful to explain the difference with a previous paper of theirs (Zappia et al 2020), in which they refer to ArgK as a “DFM marker” in the myoblast population.

Do they have corroborative evidence for “we concluded that Argk1::GFP accurately reflects the endogenous expression of Argk1”, in addition to the observed loss of GFP signal in myoblasts following Argk knockdown?

Fig 1C result: “Argk1::GFP is strongly expressed in both fusing myoblasts and multinucleated myotubes at 20 h APF, near the immature myofibrils in the myofibers at 30 h APF, and in between myofibrils in the mature muscles at 96 h APF”. Labelling these cells on the Figure, and having a single channel image for phalloidin, would help ensure that the reader can see what the authors are describing. As presented, it is not clear.

As a way of justifying their use of the two Argk RNAi lines, could the authors clarify previous characterisation of the RNAi lines they used?

The RNAi line that gives the stronger phenotype does not appear to have been used in either of the two papers Zappia et al cite as previously using ArgK lines. Zappia et al refer to the line as UAS-Argk1-RNAiJF02699 P{TRiP.JF02699}attP2. When I looked it up at BDSC, it said it was stock number 31959. Whereas the two stocks in ref 17 (Justo et el) are stock numbers 41697 and 35221.

The RNAi line that gives the weaker phenotype has been used in Bozzato et al (ref 19). However, it is not clear which line from Bozzato et al, because this paper used two VDRC lines, 34036 and 34037. I realised that the VDRC stock Zappia et al call GD10436, is in fact one of those in Bozzato et al. However, somewhat confusingly the “10436” in the paper under review is the construct ID rather than the fly line ID (either 34036 or 34037).

Fig 2: The authors “concluded that ArgK is essential throughout muscle development”.

Can the conclusion be more precise? They haven’t addressed in any detail when during development. Do they mean development of just the adult musculature, or do they mean the larval musculature also?

The main driver they use in the paper is Mef2-Gal4, which drives expression throughout embryo development (except the first few hours). Does the difference in phenotype between the Mef2Gal4 and the 1151Gal4 suggest ArgK functions in making the larval musculature, including during embryogenesis?

Muscle phenotype structure/function: Up to this point the authors have looked looking at lethality or flight function. Now they look at aspects of muscle development. Parts of this section (Figs 3 and 4) feel somewhat incomplete.

Fig 3 – They start with Mef2 Gal4 driving UAS Argk RNAi and assess the structure (including sarcomere organisation) of the flight muscles (both the IFMs and the DFMs). They quantify a decrease in cross-sectional area and myofibril width with Argk RNAi. However, there is quantification only for the IFMs.

Fig 3D – it would be helpful to have single channel phalloidin for clarity of the results.

They conclude “our findings further support the requirement of Argk1 during the development of both flight muscles”. BUT because the Argk knockdown is from so early in the developmental programme, this result tells you little about which step in the process is actually affected.

Fig 4 – They then use Act88F Gal4 to assay later in development (it is expressed from 20h after pupal formation), but with the “strong” UAS Argk RNAi line only.

They do the same muscle structure assays as with Mef2Gal4, but this time only assess the IFMs (and not the DFMs). They observe a decrease in IFM cross-section area with Argk RNAi. This appears to be less than with Mef2Gal4, but this is not commented upon.

Sarcomere length is measured here, although it doesn’t appear to have done for Mef2Gal4 in Fig 3 (although sarcomere length is measured in Fig S1 for Mef2Gal4 driving the “weaker” Argk RNAi line).

Strangely in Fig 4 is it DLM-IFM muscle 4 that is quantitated, whereas it was muscle 3 in Fig 3.

This may matter as there is evidence that the different muscles of the DLM-IFMs may not respond in the same way, e.g. see Chaturvedi et al 2019 (http://dx.doi.org/10.1098/rsob.190087)

Fig 6 scRNAseq: the Aim was to assess transcriptome effects of ArgK depletion on the wing disc associated myoblasts (they used Mef2 Gal4 and the “stronger” UAS Argk RNAi).

Their analysis divided this cell population into 8 IFM and 3 DFM groups. This is different (has more groups) to their earlier paper (Zappia et al 2020), and it would be valuable to explain the difference.

Then in a bit of a detour from the main topic for the paper, they describe various genes expressed in these defined groups, and follow one, Sp1, for the DFMs by “GTRACE”, identifying likely progenitor cells.

They then compared the AMP transcriptomes in control and Argk RNAi, and concluded that ArgK RNAi doesn’t change AMP “identity” (comparing the myoblast groups and a range of marker genes).

Fig 7 - However, a GO analysis revealed range of energy/metabolic changes in Argk knockdown. They therefore assessed ATP level, NAD+/NADH ratio and mitochondrial morphology. They did this only at the pharate stage, and with just one Gal4 driver (Mef2Gal4) and just the stronger UAS ArgK RNAi line. With Argk knockdown they found: low ATP level, altered NAD+/NADH ratio and abnormal morphology of the mitochondrial network. (n.b. Fig S3 has mitochondrial morphology results for the “weaker” UAS Argk RNAi line).

A conclusion in the Introduction is: “our work reveals a novel function of Argk1 during muscle development through its regulation of energy metabolism.” However, this seems an overstatement. Firstly, aspects of energy homeostasis are only assessed at the pharate stage, whereas the strongest developmental effects were from Argk RNAi expression driven from the earliest stages of adult muscle progenitor cells (Mef2Gal4), or others from quite young pupae (Act88F). Secondly, a causal link is not established. As it stands, it is a correlation, the authors have not clearly linked energy and muscle development. Moving beyond this would be a valuable addition.

The authors make similar comments to this in both the Abstract and the Discussion, both times saying Argk function “throughout muscle development”. Is the Argk function required throughout development, or are just at some times that have consequences later? The temporal aspect is addressed to some extent using different Gal4 drivers to induce ArgK RNAi expression, but these are not sufficient to say throughout development?

Spontaneous contractions Fig 7: “We did not detect a single twitching event in Mef2>Argk1- RNAi animals (Figure 7H, Video S2). Significantly, the animals were alive at that time point since very mild movements across all flight muscles were readily observed occasionally.” Can the authors say a bit more on this? It is obviously an important point, as they know the lethality period is sometime during pupation.

In the Discussion they comment: “we found that Argk1-depleted muscles completely lack such twitching events and, concordantly, have abnormal sarcomere structures. This finding suggests that Argk1 is required for spontaneous muscle twitching during development presumably by supporting high energy demand during contractions”. The authors could consider whether this is not the explanation and, perhaps this functional deficit might be the consequence of a prior development defect.

Minor:

Introduction paragraph 2 “muscle-specific knockdown of one of these genes, Arginine kinase 1 (Argk1), resulted in pupal lethality, ” – this needs a supporting reference.

Results: “Quantifications confirmed that the cross-sectional area of muscle 3 and 4 from DLM-IFM were statistically significantly smaller than in wild type control (Figure 3C).” Fig 3C only has muscle 3.

Results: “thus indicating that the line JF02699 had a stronger phenotype than GD10436.” – this was said already in Fig1-linked text.

Results cell size: “The wing discs from Mef2>mCherry-RNAi htl>CD8-GFP and Mef2>Argk1-RNAi htl>CD8-GFP animals were dissociated into single cell suspension” genotype is missing a “;”.

**********

**Have all data underlying the figures and results presented in the manuscript been provided?**

Large-scale datasets should be made available via a public repository as described in the *PLOS Genetics*  data availability policy, and numerical data that underlies graphs or summary statistics should be provided in spreadsheet form as supporting information.

Reviewer #1: **No:**  The raw scRNA-seq sequencing data were not provided to the reviewer

Reviewer #2: Yes

Reviewer #3: Yes

**********

PLOS authors have the option to publish the peer review history of their article (what does this mean?). If published, this will include your full peer review and any attached files.

If you choose “no”, your identity will remain anonymous but your review may still be made public.

**Do you want your identity to be public for this peer review?**  For information about this choice, including consent withdrawal, please see our Privacy Policy.

Reviewer #1: No

Reviewer #2: No

Reviewer #3: No

**Figure resubmission:**

While revising your submission, we strongly recommend that you use PLOS’s NAAS tool (https://ngplosjournals.pagemajik.ai/artanalysis) to test your figure files. NAAS can convert your figure files to the TIFF file type and meet basic requirements (such as print size, resolution), or provide you with a report on issues that do not meet our requirements and that NAAS cannot fix.

After uploading your figures to PLOS’s NAAS tool - https://ngplosjournals.pagemajik.ai/artanalysis, NAAS will process the files provided and display the results in the "Uploaded Files" section of the page as the processing is complete. If the uploaded figures meet our requirements (or NAAS is able to fix the files to meet our requirements), the figure will be marked as "fixed" above. If NAAS is unable to fix the files, a red "failed" label will appear above. When NAAS has confirmed that the figure files meet our requirements, please download the file via the download option, and include these NAAS processed figure files when submitting your revised manuscript.

**Reproducibility:**

To enhance the reproducibility of your results, we recommend that authors of applicable studies deposit laboratory protocols in protocols.io, where a protocol can be assigned its own identifier (DOI) such that it can be cited independently in the future. Additionally, PLOS ONE offers an option to publish peer-reviewed clinical study protocols. Read more information on sharing protocols at https://plos.org/protocols?utm_medium=editorial-email&utm_source=authorletters&utm_campaign=protocols

# Author comment: Author response to Decision Letter 1

**DOI:** 10.1371/journal.pgen.1012304.r002

7 Jul 2026

Attachment Submitted filename: Response to Reviewers.docx

# Peer review: Decision Letter 1

**DOI:** 10.1371/journal.pgen.1012304.r003

**Authors:** Giovanni Bosco, Lolitika Mandal

10 Aug 2026

PGENETICS-D-26-00192R1

Arginine Kinase 1 supports energy homeostasis in Drosophila flight muscle development

PLOS Genetics

Dear Dr. Frolov,

Thank you for submitting your revised manuscript to PLOS Genetics. Although the three reviewers note that there is a substantial improvement in the manuscript, they do have a few minor comments. We invite you to submit a revised version of the manuscript that addresses the points raised during the review process.

Please submit your revised manuscript by Sep 09 2026 11:59PM. If you will need more time than this to complete your revisions, please reply to this message or contact the journal office at plosgenetics@plos.org. When you're ready to submit your revision, log on to https://www.editorialmanager.com/pgenetics/ and select the 'Submissions Needing Revision' folder to locate your manuscript file.

Please include the following items when submitting your revised manuscript:

* A letter that responds to each point raised by the editor and reviewer(s). You should upload this letter as a separate file labeled 'Response to Reviewers'. This file does not need to include responses to formatting updates and technical items listed in the 'Journal Requirements' section below.

* A marked-up copy of your manuscript that highlights changes made to the original version. You should upload this as a separate file labeled 'Revised Manuscript with Track Changes'.

* An unmarked version of your revised paper without tracked changes. You should upload this as a separate file labeled 'Manuscript'.

If you would like to make changes to your financial disclosure, competing interests statement, or data availability statement, please make these updates within the submission form at the time of resubmission. Guidelines for resubmitting your figure files are available below the reviewer comments at the end of this letter.

As the corresponding author, your ORCID iD is verified in the submission system and will appear in the published article. PLOS supports the use of ORCID, and we encourage all coauthors to register for an ORCID iD and use it as well. Please encourage your coauthors to verify their ORCID iD within the submission system before final acceptance, as unverified ORCID iDs will not appear in the published article. Only the individual author can complete the verification step; PLOS staff cannot verify ORCID iDs on behalf of authors.

We look forward to receiving your revised manuscript.

Kind regards,

Lolitika Mandal, Ph.D

Academic Editor

PLOS Genetics

Giovanni Bosco

Section Editor

PLOS Genetics

Aimée Dudley

Editor-in-Chief

PLOS Genetics

Anne Goriely

Editor-in-Chief

PLOS Genetics

**Reviewers' comments:**

Reviewer's Responses to Questions

**Comments to the Authors:**

**Please note here if the review is uploaded as an attachment.**

Reviewer #1: The study of the role of Argk1 in flight muscle development by Zappia and colleagues has been substantially revised, not only with additional data, but also in a substantial rewrite to present a more logical study of the role of this important enzyme during myogenesis. They have addressed most of my concerns, especially in the explanation of what appeared to be inconsistencies in the scRNA-seq data and have made it much clearer in both the results and discussion of the different isoforms that exist for Argk1. That said, given that Z-band formation is severly affected by Mef2>Argk1 RNAi during early stages of sarcomerogenesis (Fig 3D, anti-Kettin), but unaffected by RNAi at later stages with Act88F>Argk1 RNAi (Fig 7E, anti-Kettin), this suggests that the Z-band-located shorter isoform plays an energentic role in early stages of sarcomerogenesis. While this is briefly discussed, citing the previous data, no experiments are performed eg antibody localization of the protein to the z-band, RNAi against only the longer, mitochondrial isoforms. While the manuscript is now more focussed on the mitochndrial role, this perhaps merits greater reflection and discussion. Is the mitochondrial role in energy homeostasis restricted to the flight muscle stages or is it more widespread? Knocking down Argk1 only at pharate stages and continued into adulthood through Gal80 control (eg Mef2Gal4,Gal80ts) should lead to flightless adults if this is the case.

Overall, the new data and substantial rewriting of the manuscript provide a much clearer picture of the importance of Argk1 in muscle development and the authors are more cautious in not overinterpreting their data.

Reviewer #2: I am happy to see that this revised manuscript by Zappia and colleagues now convincingly documents the localisation of Argk1 to mitochondria in myoblasts, developing myotubes and also mature flight muscle fibres. As this is now shown in Figure 1 (and not Figure 7), one can now understand the main message of the paper. The new images are beautiful!

Also, cell size was tried to assess in vivo and is shown. I agree that measuring size by FACS makes sense.

Further, biochemical background knowledge of Argk1 function was added.

Finally, mitochondria morphology is now shown throughout development with appropriate marker. The quantification, elongated vs segmented, is still not very quantitative, but the phenotype is clear, hence it would likely not add significantly to the paper to segment individual mitochondria shapes, which might be tricky in the KD.

In total, most of my suggestions have been addressed, hence I recommend publication of this manuscript.

Minor.

1. Typo in Figure 3D, one of the “phalloidin” is missing the “n”.

2. Ref 34 looked at spontaneous twitches in developing abdominal muscles and showed it is important for cross striations, which these muscles, but not the IFMs, form. To my knowledge it was not carefully investigated if the spontaneous twitches in developing IFMs until 48h APF play an instructive role in morphogenesis. There might be data on motor neuron KO but not sure if this stops the twitching. Hence, authors might want to tone down the relevance statement. Or mention the other muscles.

Reviewer #3: Due to time-pressure I am only commenting to the authors’ responses to the points I raised in my initial review.

I will comment on each of the authors’ responses in turn.

Fig1

The text on the revised p9 doesn’t have a reference for the scRNAseq results, it should have.

It is a moot point whether higher expression of ArgK in DFMs than in IFM progenitor cells makes it a “DFM marker”.

Argk1::GFP: The authors indicate that the text was revised on pages 9-10 to account for this. The meaning of the new sentence on page 11 (“Yet, the GFP signal was mainly detected in mitochondria, likely reflecting that the GFP trap line reflects the expression of the sole isoforms Argk1-A and Argk1-E”) is not clear.

Re. my Fig 1C comment on Argk1::GFP expression: I couldn’t see in the modified Fig 1 either the asked-for labelling of expression, or the single channel for phalloidin.

The authors have clarified the two VDRC ArgK RNAi stocks used as requested.

I previously commented: “The RNAi line that gives the stronger phenotype does not appear to have been used in either of the two papers Zappia et al cite as previously using ArgK lines…...”

Although the authors do not directly address my comment, they do now have a reference in the Methods to the stronger RNAi line, and no longer cite the two papers in the original submission they cited as previously using ArgK lines.

The authors have responded to my comment linked to Fig 2.

They have modified their conclusion in the original m/s that “ArgK is essential throughout muscle development” in the Abstract, p12 and Discussion. However, this does make the conclusion from this section of their work less striking.

Fig 3: the authors have added single channel phalloidin as asked for.

Fig 3: The text on new p14 says “Notably, the Argk1-depleted DFM and IFM were significantly reduced in size.”

However, there is still only quantification for (part of) the IFM, so there is no quantification for the DFMs, therefore one cannot say that the Argk1-depleted DFMs are “significantly reduced in size”. This should be modified.

Fig4: the authors have now included quantification of both muscle 3 and 4.

Fig 6 scRNAseq - the relevant section of the Results has been modified.

However, the response from the authors really doesn’t make it clear why the IFM and DFM myoblast group classification is different in their initial submitted version to PLoS Genetics when compared to their previously published classification.

It also isn’t made clear why they have now dropped their initially submitted IFM and DFM classification.

It also isn’t clear why the classification in this modified version (5 IFM groups and 1 DFM) is different to both the initially submitted version and the previously published classification.

They seem to say that the 10x Genomics sequencing methodology in this paper is more powerful than their previously used “DropSeq”.

As 10x Genomics is “more powerful”, it is not apparent why they now drop the classification of 8 IFM groups plus 3 DFM groups derived from the 10x Genomics results.

Taken together it makes one question the significance of these groups as presented.

Fig 7 comment. The authors have modified the Title, Abstract (however, it does make the conclusion from this section of their work less striking)

The narrative works better now with the energy homeostasis data moved to Figure 3 .

They have also modified the Discussion – however, on the new p30 I don’t think the new sentence at the end of the first paragraph “……. and consequently affects energy homeostasis” is really clear.

Could it not be that ArgK affects energy homeostasis and consequently aspects of the flight muscles?

The new results added to Fig 6 are helpful in aspects of ArgK effects on IFM development.

The added muscle-twitching comment is also helpful.

The minor comments are dealt with.

**********

**Have all data underlying the figures and results presented in the manuscript been provided?**

Large-scale datasets should be made available via a public repository as described in the *PLOS Genetics*  data availability policy, and numerical data that underlies graphs or summary statistics should be provided in spreadsheet form as supporting information.

Reviewer #1: Yes

Reviewer #2: Yes

Reviewer #3: Yes

**********

PLOS authors have the option to publish the peer review history of their article (what does this mean?). If published, this will include your full peer review and any attached files.

If you choose “no”, your identity will remain anonymous but your review may still be made public.

**Do you want your identity to be public for this peer review?**  For information about this choice, including consent withdrawal, please see our Privacy Policy.

Reviewer #1: No

Reviewer #2: No

Reviewer #3: No

[NOTE: If reviewer comments were submitted as an attachment file, they will be attached to this email and accessible via the submission site. Please log into your account, locate the manuscript record, and check for the action link "View Attachments". If this link does not appear, there are no attachment files.]

**Figure resubmission:**

**Reproducibility:**

To enhance the reproducibility of your results, we recommend that authors deposit laboratory protocols in protocols.io, where a protocol can be assigned its own identifier (DOI) such that it can be cited independently in the future. Additionally, PLOS ONE offers an option to publish peer-reviewed clinical study protocols. Read more information on sharing protocols at https://plos.org/protocols?utm_medium=editorial-email&utm_source=authorletters&utm_campaign=protocols

# Author comment: Author response to Decision Letter 2

**DOI:** 10.1371/journal.pgen.1012304.r004

24 Aug 2026

Attachment Submitted filename: Response_to_Reviewers_auresp_2.docx

# Editor report: Decision Letter 2

**DOI:** 10.1371/journal.pgen.1012304.r005

**Authors:** Giovanni Bosco, Lolitika Mandal

25 Aug 2026

Dear Dr Frolov,

We are pleased to inform you that your manuscript entitled "Arginine Kinase 1 supports energy homeostasis in Drosophila flight muscle development" has been editorially accepted for publication in PLOS Genetics. Congratulations!

Before your submission can be formally accepted and sent to production you will need to complete our formatting changes, which you will receive in a follow up email. Please be aware that it may take several days for you to receive this email; during this time no action is required by you. Please note: the accept date on your published article will reflect the date of this provisional acceptance, but your manuscript will not be scheduled for publication until the required changes have been made.

Once your paper is formally accepted, an uncorrected proof of your manuscript will be published online ahead of the final version, unless you’ve already opted out via the online submission form. If, for any reason, you do not want an earlier version of your manuscript published online or are unsure if you have already indicated as such, please let the journal staff know immediately at plosgenetics@plos.org.

In the meantime, please log into Editorial Manager at https://www.editorialmanager.com/pgenetics/, click the "Update My Information" link at the top of the page, and update your user information to ensure an efficient production and billing process. Note that PLOS requires an ORCID iD for all corresponding authors. Therefore, please ensure that you have an ORCID iD and that it is validated in Editorial Manager. To do this, go to ‘Update my Information’ (in the upper left-hand corner of the main menu), and click on the Fetch/Validate link next to the ORCID field.  This will take you to the ORCID site and allow you to create a new iD or authenticate a pre-existing iD in Editorial Manager.

If you have a press-related query, or would like to know about making your underlying data available (as you will be aware, this is required for publication), please see the end of this email. If your institution or institutions have a press office, please notify them about your upcoming article at this point, to enable them to help maximise its impact. Inform journal staff as soon as possible if you are preparing a press release for your article and need a publication date.

Thank you again for supporting open-access publishing; we are looking forward to publishing your work in PLOS Genetics!

Yours sincerely,

Lolitika Mandal, Ph.D

Academic Editor

PLOS Genetics

Giovanni Bosco

Section Editor

PLOS Genetics

Aimée Dudley

Editor-in-Chief

PLOS Genetics

Anne Goriely

Editor-in-Chief

PLOS Genetics

www.plosgenetics.org

BlueSky: @plos.bsky.social

----------------------------------------------------

Comments from the reviewers (if applicable):

----------------------------------------------------

**Data Deposition**

If you have submitted a Research Article or Front Matter that has associated data that are not suitable for deposition in a subject-specific public repository (such as GenBank or ArrayExpress), one way to make that data available is to deposit it in the Dryad Digital Repository. As you may recall, we ask all authors to agree to make data available; this is one way to achieve that. A full list of recommended repositories can be found on our website.

The following link will take you to the Dryad record for your article, so you won't have to re‐enter its bibliographic information, and can upload your files directly:

http://datadryad.org/submit?journalID=pgenetics&manu=PGENETICS-D-26-00192R2

More information about depositing data in Dryad is available at http://www.datadryad.org/depositing. If you experience any difficulties in submitting your data, please contact help@datadryad.org for support.

Additionally, please be aware that our data availability policy requires that all numerical data underlying display items are included with the submission, and you will need to provide this before we can formally accept your manuscript, if not already present.

----------------------------------------------------

**Press Queries**

If you or your institution will be preparing press materials for this manuscript, or if you need to know your paper's publication date for media purposes, please inform the journal staff as soon as possible so that your submission can be scheduled accordingly. Your manuscript will remain under a strict press embargo until the publication date and time. This means an early version of your manuscript will not be published ahead of your final version. PLOS Genetics may also choose to issue a press release for your article. If there's anything the journal should know or you'd like more information, please get in touch via plosgenetics@plos.org.

# Editor report: Acceptance letter

**DOI:** 10.1371/journal.pgen.1012304.r006

**Authors:** Giovanni Bosco, Lolitika Mandal

PGENETICS-D-26-00192R2

Arginine Kinase 1 supports energy homeostasis in Drosophila flight muscle development

Dear Dr Frolov,

We are pleased to inform you that your manuscript entitled "Arginine Kinase 1 supports energy homeostasis in Drosophila flight muscle development" has been formally accepted for publication in PLOS Genetics! Your manuscript is now with our production department and you will be notified of the publication date in due course.

The corresponding author will soon be receiving a typeset proof for review, to ensure errors have not been introduced during production. Please review the PDF proof of your manuscript carefully, as this is the last chance to correct any errors. Please note that major changes, or those which affect the scientific understanding of the work, will likely cause delays to the publication date of your manuscript.

Soon after your final files are uploaded, unless you have opted out or your manuscript is a front-matter piece, the early version of your manuscript will be published online. The date of the early version will be your article's publication date. The final article will be published to the same URL, and all versions of the paper will be accessible to readers.

For Research Articles, you will receive an invoice from PLOS for your publication fee after your manuscript has reached the completed accept phase. If you receive an email requesting payment before acceptance or for any other service, this may be a phishing scheme. Learn how to identify phishing emails and protect your accounts at https://explore.plos.org/phishing.

Thank you again for supporting PLOS Genetics and open-access publishing. We are looking forward to publishing your work!

With kind regards,

Janani Seenivasan

PLOS Genetics

On behalf of:

The PLOS Genetics Team

Carlyle House, Carlyle Road, Cambridge CB4 3DN | United Kingdom

plosgenetics@plos.org | +44 (0) 1223-442823

plosgenetics.org | Twitter: @PLOSGenetics
